# Linking stem growth respiration to the seasonal course of stem growth and GPP of Scots pine

**DOI:** 10.1093/treephys/tpy040

**Published:** 2018-05-16

**Authors:** Tommy Chan, Frank Berninger, Pasi Kolari, Eero Nikinmaa, Teemu Hölttä

**Affiliations:** 1 Institute for Atmospheric and Earth System Research/Forest Sciences, Faculty of Agriculture and Forestry, University of Helsinki, PO Box 27, Helsinki, Finland; 2 Institute for Atmospheric and Earth System Research/Physics, Faculty of Science, University of Helsinki, PO Box 68, Helsinki, Finland

**Keywords:** dendrometers, eddy covariance, GPP, growth, growth efficiency, radial variations, respiration

## Abstract

Current methods to study relations between stem respiration and stem growth have been hampered by problems in quantifying stem growth from dendrometer measurements, particularly on a daily time scale. This is mainly due to the water-related influences within these measurements that mask growth. A previously published model was used to remove water-related influences from measured radial stem variations to reveal a daily radial growth signal $\left(\hat{\Delta }G_{m}\right)$. We analysed the intra- and inter-annual relations between $\hat{\Delta }G_{m}$ and estimated growth respiration rates (*R*_g_) on a daily scale for 5 years. Results showed that *R*_g_ was weakly correlated to stem growth prior to tracheid formation, but was significant during the early summer. In the late summer, the correlation decreased slightly relative to the early summer. A 1-day time lag was found of $\hat{\Delta }G_{m}$ preceding *R*_g_. Using wavelet analysis and measurements from eddy covariance, it was found that *R*_g_ followed gross primary production and temperature with a 2 and 3 h time lag, respectively.This study shows that further in-depth analysis of in-situ growth and growth respiration dynamics is greatly needed, with a focus on cellular respiration at specific developmental stages, its woody tissue costs and linkages to source–sink processes and environmental drivers.

## Introduction

Stem growth, net primary production (NPP) and the physiological costs to maintain and grow woody tissue have been of great interest because of their contribution to forest carbon budgets. In particular, the components of NPP, gross primary production (GPP) and autotrophic respiration have been studied extensively in relation to biomass growth and carbon budgets (Ryan et al. 1997, Xiao et al. 2003, Goulden et al. 2011). In relation to stand or ecosystem energy budgets, autotrophic respiration, for example, can consume anywhere from 30% to 70% of the total carbon fixed for maintenance and tissue construction (Sprugel and Benecke 1991, Ryan et al. 1997). Usually, respiration is studied at the ecosystem level with the eddy covariance technique, which can estimate both GPP and ecosystem respiration (which includes autotrophic and heterotrophic components) (Desai et al. 2008). Yet the contribution of different tree organs to ecosystem respiration and individual trees is unclear, particularly as methodologies for measuring stem respiration and stem growth differ. This ultimately produces substantially varying results.

A common method for measuring stem respiration is with an open flow-through chamber installed onto a stem segment. By calculating the difference between the carbon dioxide (CO_2_) concentration entering and exiting the chamber, the stem CO_2_ efflux (*E*_S_) can be determined. Since *E*_S_ from a stem segment is the measured gas exchange between woody stem and external atmosphere, it is often associated with the rate of physiologically active respiration of the living tissues (i.e., phloem, cambium and xylem parenchyma) (Saveyn et al. 2008). However, the relationship between *E*_S_ and actual respiration is difficult to assess, as CO_2_ is carried to and from the point of measurement vertically along the xylem (Teskey and McGuire 2002, Teskey et al. 2008, Hölttä and Kolari 2009, Bloemen et al. 2013).

One approach to quantifying respiration, with respect to stem carbon budgeting, is by separating *E*_S_ into two functional components: respiration costs used to build new dry matter (growth respiration) and respiration needed to maintain the life processes of living cells (maintenance respiration) (Amthor 2012). By estimating either the growth or maintenance respiration component first, the difference between total respiration and the estimate will reveal the other component. Various studies have estimated the growth component first, revealing the maintenance component as the difference (Ryan et al. 1994, Carey et al. 1996), but estimating the maintenance component first remains the most widely preferred method (Stockfors and Linder 1998, Maier 2001). One method to separate total stem CO_2_ efflux into maintenance respiration and growth respiration components is the mature tissue method (Amthor 2012). This method assumes that the total respiration from mature tissue (i.e., tissue that is no longer growing) may be attributed to the maintenance respiration component. Thus, calculating maintenance respiration rates outside of the growing season (typically before day of year (DOY) 130 and after DOY 240) can be used to estimate annual maintenance respiration rates.

Separating *E*_S_ into these components allows for further investigation of stem carbon budgeting and tissue costs. However, this separation requires caution—estimation of maintenance and growth respiration rates may not be wholly independent of one another (Lavigne et al. 1996). Therefore, it can be difficult to correctly separate the two components. In addition, the growth respiration derived from the mature tissue method has a tendency to be estimated higher compared with other theoretical methods (Sprugel and Benecke 1991), suggesting that the products of growth respiration are more demanding—and possibly that all woody respiration during the growing season is solely related to growth regardless of is partitioning.

It has been reported that both external and internal drivers regulate *E*_S_ (Maier et al. 2010). For example, studies have extensively documented influences from water (Rodríguez-Calcerrada et al. 2014, Salomón et al. 2016), temperature (Stockfors and Linder 1998, Zha et al. 2004, Gruber et al. 2009, Brito et al. 2010, Reich et al. 2016), sap flow (Negisti 1972, Negisi 1975, Bowman et al. 2005, Gansert and Burgdorf 2005), carbohydrate supply (Maunoury-Danger et al. 2010) and the phenological phases of the tree (Asshoff et al. 2006). In principle, the growth respiration component of *E*_S_ is correlated with stem growth with a short time lag (Ryan 1990). However, previous studies have reported inconsistent results. For example, studies correlating stem growth rate (SGR) and stem growth respiration rates (*R*_g_) showed a lag range varying from 0 to 40 days (Zumer 1969, Edwards and Hanson 1996, Zha et al. 2004). One factor that may contribute to these large differences is the measurement technique. Stem growth and *R*_g_ correlation studies mainly utilize dendrometers to measure stem radius/diameter increment—a proxy for volumetric stem growth. However, it is clear that growth taken directly from these instruments also includes water-related changes (Daudet et al. 2005, Mencuccini et al. 2013, Chan et al. 2016, Zweifel 2016). These changes (although reversible and small on a seasonal scale) are typically larger than growth on a daily scale, and can therefore mask quantifiable short-term (e.g., daily) growth. Studies have circumvented this problem by using dendrometer measurements on a bi-weekly (Maier 2001), monthly (Vose and Ryan 2002, Zha et al. 2004) and annual (Araki et al. 2015) scale. However, the consequence is the loss of daily dynamics and the possibility to link *R*_g_ directly to SGR or environmental conditions. It is, therefore, important to analyse growth on a daily scale in order to understand respiration dynamics throughout the year, especially during the growing season.

It has been suggested that *R*_g_ is proportional to SGR, while temperature should not affect it apart from influencing the SGR (Lavigne and Ryan 1997, Loveys et al. 2002, Vose and Ryan 2002). Additionally, changes in chemical composition of the wood can affect *R*_g_ by increasing or decreasing construction cost. As woody tissue progresses through different stages of physiological or biochemical changes during the growing season, it is possible to explain whether *R*_g_ varies in relation to these different stages (Vose and Ryan 2002). This has been supported in a study at the ecosystem level, where the intra-annual changes of GPP and *E*_S_ were highly correlated (Zha et al. 2004). Stem growth undergoes three predominant processes before maturation: cell division, cell enlargement and cell wall thickening/deposition (Rossi et al. 2006*a*). Growth derived from dendrometers show only the influence of cell division and enlargement processes to tissue dimension, whereas growth derived from microcores can identify all three processes (Rossi et al. 2006*a*, Deslauriers et al. 2007, Mäkinen et al. 2008). On the other hand, carbon-fixation observed from eddy covariance measurements refers to the difference between ecosystem photosynthesis and respiration over a given time period in a given area, and thus cannot be used to observe cellular growth (Pretzsch 2009). However, as GPP also provides resources for both growth and respiration analysis, comparison with tree-level growth respiration dynamics may also prove valuable. By separating growth into distinct phases, each representing the predominant growth process, intra-annual *R*_g_ to growth dynamics can be studied. This has been shown in previous studies, but only on specific days (Stockfors and Linder 1998) and on a monthly scale (Vose and Ryan 2002). When exactly these correlations appear and what metabolic processes occur during these times have yet to be further explored.

In this study, we investigate the relationship and dynamics between GPP, growth and growth respiration on a daily time scale to provide a physiological understanding of tree growth. We link this relationship to whole year and apparent (i.e., intra-annual) growth efficiency by observing the year’s biomass growth. We also relate derived estimates of GPP from eddy covariance measurements to the study. The SGR was estimated using a previously published model (Chan et al. 2016) on field-measured dendrometer data of *Pinus sylvestris* L. (Scots pine). We hypothesize that the dynamics between growth, growth respiration and GPP can be observed during the whole year if we apply the model to separate the growth component from dendrometer measurements. Our objectives were: (i) to compare the dynamics of *R*_g_ with SGR derived from the corrected water-related model estimate and direct (i.e., raw) radial stem measurements at different growth phases; (ii) to identify the time lag between *R*_g_ and SGR; (iii) to calculate the annual budget for woody-tissue respiration; and (iv) compare the dynamics of *R*_g_ to both GPP and temperature using wavelet analysis.

## Materials and methods

### Site description

The study site is located at SMEAR II (System for Measuring Forest Ecosystem-Atmosphere Relationships II) research station in Hyytiälä, Finland (61°51′N, 24°17′E, 170 m above sea level). The study site is an even-aged, 51-year-old Scots pine forest, belonging to the southern boreal zone. The forest area is of medium site quality (Vaccinium-type) according to the Cajander class system (Cajander 1909) (see Vesala et al. (2005) for detailed description). The mean height of the stand is 17.4 m with a mean diameter at breast height (1.3 m) of 18 cm, with a typical stemwood growth rate of 8 m^3^ ha^−1^ year^−1^ (2014 data). The yearly average GPP, TER (total ecosystem respiration) and NEE (net ecosystem CO_2_ exchange) during the growing period (April–October; 1997–2014) from eddy covariance measurements over the stand is 5.34, 3.41 and 1.92 μmol m^−2^ s^−1^, respectively (refer to Table 1 for the main terminology used). The parent material of the soil is composed of sandy and coarse silty glacial till and the soil is a Haplic Podzol. The mean annual precipitation of the area is 713 mm, with a mean annual air temperature of 3.3 °C. The warmest and coldest months are July (mean +15.3 °C) and January (mean −8.9 °C), respectively. The growing season generally begins in late April and ends around early September.

**Table 1.** A summary of the terminology and definitions used.

Terminology	Definition
*E*s	Measured stem CO_2_ efflux (μg CO_2_ m^−2^ s^−1^)
*R*_g_	Growth respiration rate (μg CO_2_ m^−2^ s^−1^)
*R*_m_	Maintenance respiration rate (μg CO_2_ m^−2^ s^−1^)
SGR	Stem growth rate
GPP	Gross primary production (μmol m^−2^ s^−1^)
$\hat{\Delta }G_{m}$	Modelled growth; model-estimated accumulated growth during a season—i.e., estimated cambial growth and change of radius due to osmotic concentration variation (mm)
$\Delta \hat{\Delta }G_{m}$	Modelled daily growth rate; daily derivative of $\hat{\Delta }G_{m}$, representing the daily estimated growth rate (mm day^–1^)
*D*_w_	Raw growth; measured whole-stem radial variation (mm)
*ΔD*_w_	Raw daily growth rate; daily derivative of *D*_w_, represented by the daily growth rate (mm day^–1^)
*D*_b_	Measured inner-bark radial variation (mm)
*D*_x_	Measured xylem radial variation (mm)
*Q*_10_	Temperature coefficient of respiration
*Y*	Growth efficiency
CWT	Continuous wavelet transform
WC	Wavelet coherence

### Environmental variables and stand level fluxes

Year-round field data of air temperature (°C) was recorded continuously from a tower at a height of 8.4 m at 1-min intervals and measured with radiation-shielded pt-100-type resistance thermometer sensors. The estimate of GPP was derived from eddy covariance measurements of NEE. Gross primary production (μmol m^−2^ s^−1^) was calculated by subtracting a temperature-driven model of TER from the measured NEE (see Kolari et al. (2009) for further details). The model was based on a periodic temperature response from night-time measurements. In total, five trees were used in the study (see Table 2): three trees were measured continuously from 2007 to 2009 and in 2011, and two trees in 2015. Of the trees monitored from 2007 to 2009 and in 2011, in one tree both radial stem variations and stem CO_2_ efflux were measured, while in the remaining two only radial stem variations were measured. To distinguish the trees measured in 2015, one tree was denoted as 2015a and the other as 2015b. In consideration of the fact that the stem CO_2_ efflux chamber was installed on one tree for this study from 2007 to 2011, further data analysis was performed from that tree only, and dendrometer measurements from the other trees were used to validate modelled growth (see Figure S1 available as Supplementary Data at *Tree Physiology* Online).

**Table 2.** The number of trees monitored in the study for each year, and the type of measurements performed on each tree. Statistical analysis was performed on trees 1, 4 and 5. Trees 2 and 3 were used to corroborate the growth model. In the current study, trees 4 and 5 are denoted as 2015a and 2105b, respectively.

Year	Tree no.	Measurements
2007–09, 2011	1	Radial stem variations, stem CO_2_ efflux
	2	Radial stem variations
	3	Radial stem variations
2015	4	Radial stem variations, stem CO_2_ efflux
	5	Radial stem variations, stem CO_2_ efflux

### Dendrometer measurements

Radial stem variations, expressed in mm, were measured using two LVDT dendrometers (linear variable-displacement transducers; model AX/5.0/S, Solartron Inc., West Sussex, UK) at a height of ~15 m, below the canopy. Dendrometers were installed on three trees for years 2007–09 and 2011, and on two trees for year 2015. The dendrometers were connected to a data logger (21×, Campbell Scientific Ltd, Leics, UK) by means of multiplexers (SDMX50, Campbell Scientific Ltd) and measured continuously at 1-min intervals. The dendrometers were secured to a rectangular stainless-steel frame (Sandvik 1802 Steel, Sandvik, Sandvixen, Sweden) and spaced 30 mm apart. The frame was then affixed onto the tree with screws using attachment plates (Sevanto et al. 2005). One dendrometer measured xylem radial variation (*D*_x_). The head of this dendrometer rested against a small screw that was inserted ~10 mm through the outer- and inner-bark, into the superficial part of the existing xylem. The second dendrometer measured whole-stem radial variation (*D*_w_), which rested on the phloem. The phloem was exposed by incising ~3 mm of the outer-bark with a scalpel (see Chan et al. (2016) for further details). This study used *D*_x_ and inner-bark radial variation (*D*_b_). *D*_b_ was calculated as the difference between *D*_w_ and *D*_x_. *D*_b_, then, includes the phloem tissue produced to the outside of the pre-existing xylem tissue, the vascular cambium and newly formed xylem.

### Estimation of radial stem growth

Radial stem variation arises due to three main processes: irreversible cambial growth, a reversible xylem water potential-induced change and a reversible osmotic concentration change (Sevanto et al. 2003, Daudet et al. 2005, Mencuccini et al. 2013). We separated the transpiration-driven, xylem water potential-induced change (derived from xylem radial variation) from measured inner-bark (derived from dendrometer data), to reveal the variations caused by osmotic concentration and cambial growth (Chan et al. 2016). This approach applies Hooke’s Law, where water-related changes in the xylem radius reflected changes in xylem water potential (Perämäki et al. 2001). Accordingly (in the absence of any other influences), the inner-bark would also follow xylem water-related changes, tending towards equilibrium. Therefore, the predicted change of the measured inner-bark radius $\left(\hat{\Delta }D_{b}\right)$ that is solely affected by xylem water potential, at time (*t* + *Δt*) (i.e., the next measuring point), can be predicted from the changes in inner-bark and xylem radii at time (*t*) (Mencuccini et al. 2013, Chan et al. 2016):

(1) $$\hat{\Delta }D_{b}\left(t+\Delta t\right)=\hat{\Delta }D_{b}\left(t\right)+\alpha \left(\beta \Delta D_{x}\left(t\right)-\hat{\Delta }D_{b}\left(t\right)\right)\Delta t,$$

where $\Delta D_{x}$ is the measured radial change in xylem radius, *α* is the radial hydraulic conductance between the xylem and inner-bark and *β* is the ratio of the elastic modulus of the inner-bark to xylem (for a given change in xylem potential). Parameters *α* and *β* were estimated by employing a non-linear, least-square regression fitting over Eq. (1) using inner-bark and xylem measurements at 30-min intervals, over the whole sampling period (Excel Solver, Microsoft, USA). The difference between the measured inner-bark radius $\left(\Delta D_{b}\right)\;$ and $\hat{\Delta }D_{b}$ is the variation that is not explained by the xylem water potential $\left(\hat{\Delta }G_{m}\right)$:

(2) $$\hat{\Delta }G_{m}\left(t\right)=\Delta D_{b}\left(t\right)-\hat{\Delta }D_{b}\left(t\right).$$

In other words, $\hat{\Delta }G_{m}$, expressed in mm, can be used as proxy for volumetric radial stem growth. In reality, $\hat{\Delta }G_{m}$ also includes influences from reversible osmotic concentration changes of the inner-bark, which we were not able to further separate. However, our previous analysis suggested that the osmotic concentration change effect on $\hat{\Delta }G_{m}$ would maximally be ~30% on a diurnal scale (Chan et al. 2016). For additional detail in modelling estimation and parameterization, refer to Chan et al. (2016).

### Calculation of growth efficiency

The growth efficiency (*Y*) is a dimensionless coefficient defined as the ratio at which sugar is transformed into new dry mass (*ΔW*) to the total amount of assimilates required for this transformation to occur, which consists of the sum of the total accumulated growth respiration (*R*_ga_) and *ΔW* of the stem (Thornley 1970, Roux et al. 2001):

(3) $$Y=\frac{\Delta }{\left(\Delta W+R_{ga}\right)}.$$

Growth efficiency is apparent on a time scale shorter than annual, since the observed growth dynamics are due to the change in volume growth and not accumulated mass. In this study, the accumulated mass (in terms of *Y*) is of interest, and at the annual scale, the volume and accumulated mass should be linearly proportional to one another, provided that wood density stays constant (which is assumed). However, it is expected that biomass accumulation continues after radial width increment ceases, and likely at a higher rate due to the accumulation of thick-walled latewood (Cuny et al. 2015).

*Δ* *W* is observed as the amount of carbon content-per-wood volume per unit length $\left(\rho_{\frac{carbon_{content}}{wood_{volume}}}\right)$ within a certain change in volume of stem per unit length (*ΔV*):

(4) $$\Delta W=\rho_{\frac{carbon_{content}}{wood_{volume}}}\Delta V.$$

For Scots pine, it is estimated that $\rho_{\frac{carbon_{content}}{wood_{volume}}}$ is ~220 kg m^−3^. *ΔV* can further be expressed as

(5) ΔV≈2πrΔd≈πdΔd,

where *d* is the diameter of the tree and *Δd* is maximum $\hat{\Delta }G_{m}$ after the growing season. From this equation, Eq. (6) can be rewritten to express the change in volume as the change in inner-bark diameter (for 1 m length of stem):

(6) $$\Delta W=\rho_{\frac{carbon_{content}}{wood_{volume}}}\pi d\Delta d.$$

For estimation of *Y*, we assume that developing wood has the same density as mature wood. Although the intra-annual density between earlywood and latewood varies, the relative difference is subtle (Jyske et al. 2008). *Y* was calculated annually and for the second and third phases of each year (see Statistical analysis section for phase separation). It was not calculated for the first phase since appreciable radial growth did not yet readily occur. Due to insufficient *E*_S_ data for one tree in 2015 (~41 days missing), calculation of *Y* for this tree was not possible.

### Stem CO_2_ efflux measurements

Stem CO_2_ efflux (*E*_S_), expressed as μg CO_2_ m^−2^ s^−1^, was measured using an automated chamber system comprising of an acrylic chamber (height 20 cm and width 3.5 cm) attached to the bark of the tree (Kolari et al. 2009). A continuous flow of 1.3 l min^–1^ through the system was applied to measure stem CO_2_ efflux. Within the chamber, the efflux was measured as the difference between the CO_2_ concentration of the ambient air into the system and the CO_2_ concentration flowing out from the system. The chamber system was installed at a height of 12 m in 2007 and moved to 11.4 m (25 June) in 2008 due to technical maintenance. In 2009, measurement height was at 14.1 m. In 2011 (13 July), the continuous flow was adjusted to 1.1 l min^–1^ due to further technical diagnostics.

Measured *E*_*S*_ does not wholly represent all locally respired CO_2_ (Trumbore et al. 2013). It could differ due to (i) the release of CO_2_ from other parts of the tree that has been transported upwards in xylem water; (ii) the removal of CO_2_ by the transpiration stream; and (iii) the release of CO_2_ in xylem storage pools (Hölttä and Kolari 2009, Ubierna et al. 2009). To limit the influence of (i) and (ii) for this study, night-time stem respiration rates (22:00–05:00 h) were used in order to capture CO_2_ efflux that had not been significantly affected by axial convection of respired CO_2_ along with xylem sap flow. Although sap flow may occur during the night, it has been shown to be small (Daley and Phillips 2006). The contribution from the release of CO_2_ in xylem storage pools to measured *E*_S_ was found to be negligible (McGuire and Teskey 2004, Saveyn et al. 2008) and thus omitted in this study.

An exponential equation was used to describe the temperature response of woody tissue respiration (*R*):

(7) $$R=E_{S10}\times {Q_{10}}^{(10-T)/10},$$

where *E*_S10_ is the CO_2_ efflux at a tissue temperature of 10 °C, *Q*_10_ is the temperature coefficient of respiration and *T* is the lagged temperature at reference point *R*. Studies have found an acclimation response of respiration to the seasonal variation in temperature (Atkin and Tjoelker 2003). In this study, respiration followed temperature with a time lag of no more than 2 h, which is due to a diffusion resistance to movement of CO_2_ from the stem to air (Ryan et al. 1995, Lavigne et al. 1996). To compensate for this lagged response; respiration was regressed against prior temperature of 1.5 h as it gave the best fit.

To calculate annual maintenance respiration rates (*R*_m_), *Q*_10_ was calculated over a continuous interval of 2 weeks during a period of non-growth activity (i.e., 2 weeks before DOY 130 or 2 weeks after DOY 240). It is assumed that during this period, *E*_S_ was caused solely by the maintenance respiration component, and that changes due to temperature would also reflect similar changes during the growing season. Thus, *Q*_10_ and *E*_S10_ parameter values derived from the non-growing period were used with Eq. (3) to estimate *R*_m_ (μg CO_2_ m^−2^ s^−1^).

*Q*_10_ was calculated separately for each dataset using the following equation and the value obtained was inserted into Eq. (3):

(8) $$Q_{10}={\left(\frac{R_{2}}{R_{1}}\right)}^{\frac{10}{T_{2}-T_{1}}},$$

where *R*_1_ and *R*_2_ are CO_2_ efflux rates at temperatures *T*_1_ and *T*_2_. Growth respiration rate (*R*_g_; μg CO_2_ m^−2^ s^−1^) was then calculated as the difference between *R*_m_ and measured total stem CO_2_ efflux (*E*_S_).

### Statistical analysis

To fully capture the summer growing season, this study used 30-min mean values from 1 April to 5 October for the years 2007–09, 2011 and 2015. Field-collected radial stem variations and $\hat{\Delta }G_{m}$ were set to zero on 1 April of each year as this date precedes the beginning of the growing season. Intra-annual tree growth was divided into three distinct phases: the first phase was defined as the period from 1 April to the date when the first tracheids were observed from microcores (8 June, 29 May and 28 May in 2007, 2008 and 2009, respectively; refer to Chan et al. (2016) for reference and microcore sampling method). No microcore samples were taken in 2011 and 2015, so an estimate was made based on observations from previously sampled years as to when the first tracheids may have been formed. The second phase was defined as the period from the end of the first phase to the date when a significant decline of SGR occurred, based on a calculated moving average of 10 days (25 July, 7 August, 2 August, 1 August and 20 July for years 2007, 2008, 2009, 2011 and 2015, respectively) derived from dendrometers. The third phase was defined as the period from the end of the second phase to 5 October.

For some analyses, daily mean values were calculated for *R*_g_, temperature and $\hat{\Delta }G_{m}$. In addition, the daily derivatives of $\hat{\Delta }G_{m}$ ($\Delta \hat{\Delta }G_{m}$; mm day^–1^) and raw whole-stem radial variation (*ΔD*_w_; mm day^–1^) were calculated (i.e., the difference between two consecutive daily maximum values) and represented as daily SGR. Cross-correlation analysis was performed for each year between daily *R*_g_ to each daily SGR series to reveal any time lag, and adjusted to this lag when performing coefficient of determination analyses (*r*^2^) between *R*_g_ to each daily SGR series. All *r*^2^ analyses were performed using linear regression (SPSS v23). For manuscript readability, $\hat{\Delta }G_{m}$ will hereafter be called modelled growth (accumulated growth over the whole season); $\Delta \hat{\Delta }G_{m}$, modelled daily growth (daily growth rate); *ΔD*_w_, raw growth (accumulated growth over the whole season, the raw dendrometer signal); and $\Delta \Delta D_{w}$, raw daily growth (daily growth rate, the raw dendrometer signal). Since partitioning respiration into growth and maintenance respiration component is not unambiguous and straightforward (e.g., Ryan et al. 2009), we also conducted a parallel analysis (see Supplementary Data at *Tree Physiology* Online) where we did not partition respiration into growth vs maintenance respiration, but simply compared growth against respiration, i.e., using *E*_S_ in place of *R*_g_.

To compare the temporal variability in the relationship between *R*_g_ to both temperature and GPP, we used wavelet analysis, specifically continuous wavelet transform (CWT) and wavelet coherence (WC) analysis using the wavelet toolbox (Grinsted et al. 2004) in Matlab R2016a. Previous studies have described wavelet analysis in detail (Torrence and Compo 1998, Grinsted et al. 2004, Vargas et al. 2011, Gea-Izquierdo et al. 2015, Natalini et al. 2016). Continuous wavelet transform identifies the common period in the time–frequency space. Meanwhile, WC analysis identifies the coherence (high and low)—i.e., the measured linear dependency between two series, by testing for similar frequency components (Torrence and Compo 1998). Wavelet coherence analysis is used because it allows for detection of phase differences (i.e., time lags) and its variation between the two series over time, and thus, time lags can be determined at each period where high coherence is found (Vargas et al. 2011). For each wavelet analysis, the 5% significance level and edge effects were calculated.

## Results

### Measured and modelled radial growth sampling

**Figure 1.**
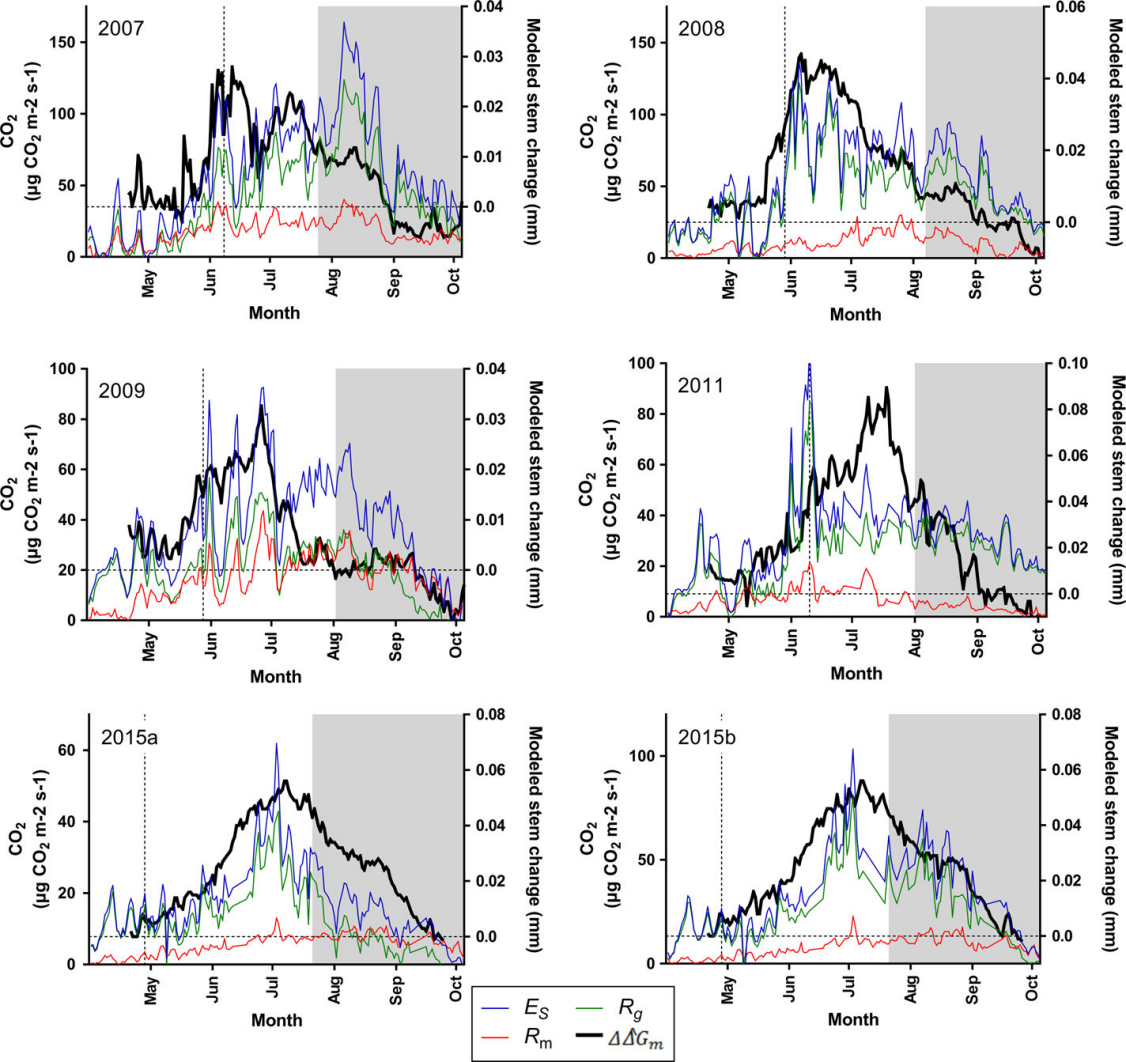
Daily intervals of stem CO_2_ efflux (*E*_S_, blue), maintenance respiration (*R*_m_, red) and growth respiration (*R*_g_, green). The dotted, vertical black line denotes the first observation of tracheid formation (i.e., beginning of the second phase) from measured microcores. The dotted, horizontal line denotes the zero point of the daily growth rate derived from the model $\left(\Delta \hat{\Delta }G_{m}\right)$. $\Delta \hat{\Delta }G_{m}$ (black) was averaged over 10 days to determine the third phase (grey shade). However, all correlation analyses in this study used daily (non-averaged) values.

Measured xylem radial variation (*D*_x_) showed a consistent diurnal pattern during the course of the growing season for all years – shrinking due to transpiration during the day, and swelling during the night without any major change in the minimum and maximum values (refer to Figure 4 in Chan et al. (2016)). Raw growth (*D*_w_) over the same time course had a similar diurnal pattern, and on a seasonal scale, a sigmoidal increase. This increase exhibited an exponential rise of the daily maximum value in the early summer, a phase of linear increase after mid-July, and reaching its seasonal maximum radius in mid-August before declining slightly shortly after in late September (not shown). As expected, modelled growth showed a high correlation to raw growth in all years over the whole growing season (not shown)—modelled growth began approximately at the same time as raw growth increment (week 20/21) and reached its seasonal peak in growth in late July/early August. Water-related changes, as seen in raw growth, masked observable growth on the sub-diurnal scale and on a diurnal scale during (and shortly after) precipitation events. During these precipitation events, the stem swelled considerably until the rains had subsided. Modelled daily growth $\left(\Delta \hat{\Delta }G_{m}\right)$ increased from late May to late June, when rapid development of stem growth occurred in all years (Figure 1; see Figure S1 available as Supplementary Data at *Tree Physiology* Online). After this rapid increase, modelled daily growth declined rapidly at the beginning of July from years 2007 to 2009 and 2015, and on 20 July in 2011. After August, modelled daily growth decline was further pronounced, with a SGR below zero at the end of the measurement period. Remarkably, the patterns of modelled daily growth of the two trees in 2015 were very similar.

### Relation of stem CO_2_ efflux to stem growth

**Figure 2.**
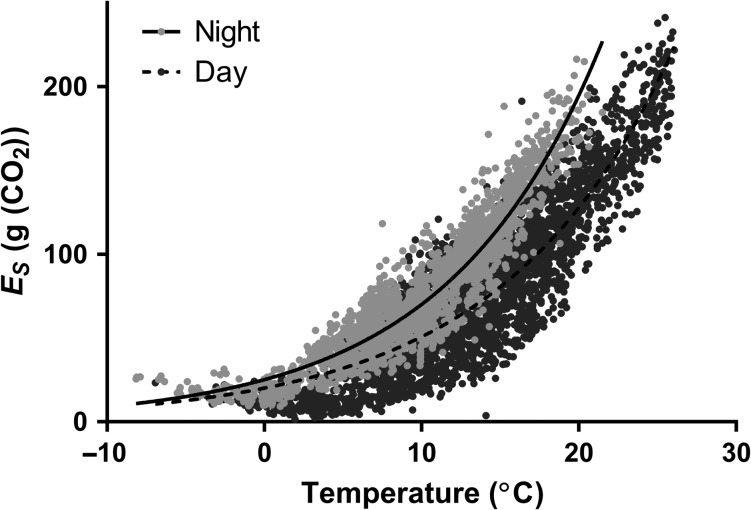
Regression between stem CO_2_ efflux (*E*_S_) and temperature during the day (07:00–19:00 h, dark grey) and night (22:00 and 05:00 h, light grey) at half-hourly intervals with their respective exponential fitted curve from 1 April to 5 October 2007. Figure has been corrected with a 1.5 h temperature lag to *E*_S_.

Observed stem CO_2_ efflux rates (*E*_S_) had a close exponential relationship with air temperature throughout the study period. During the night (22:00–05:00 h), *E*_S_ and temperature correlations showed a year-to-year *r*^2^ range from 0.62 to 0.85, and had a considerably higher *r*^2^ than day-time values (07:00–19:00 h), which ranged from 0.59 to 0.80 (Figure 2). As in typical boreal environments, periods before May and after September had lower *Q*_10_ values (1.31–1.46) than summer (June–August) *Q*_10_ values (1.92–2.07).

Generally, *R*_m_ was much smaller and less variable in comparison with *R*_g_ over the whole growing season, in all years (Figure 1). *R*_g_ corresponded with changes in *E*_S_, with transient growth respiration activity already occurring as early as the first week of April. In late May, *R*_g_ increased rapidly in comparison with *R*_m_, and maintained this high rate until late August. In late August, *R*_g_ began to decline steadily to springtime levels.

**Figure 3.**
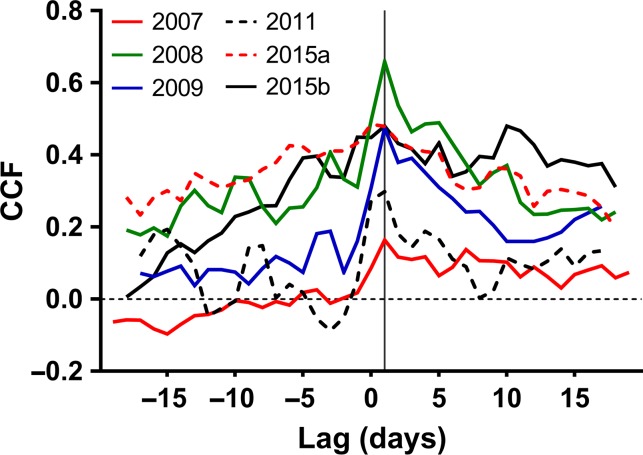
Cross-correlation function (CCF) of daily-averaged, night-time values of growth respiration (*R*_g_) to modelled daily growth rate $\left(\Delta \hat{\Delta }G_{m}\right)$ for all years studied. Dotted horizontal line indicates zero point. Vertical solid line indicates period with highest correlation.

To correlate the daily SGR series (i.e., modelled daily growth and raw daily growth) to *R*_g_, the daily mean values of *R*_g_ were regressed against each daily SGR series and separated into three growth phases: first, second and third (Figure 1). A cross-correlation function of *R*_g_ to each daily SGR series covering the second and third phases revealed a 1-day time lag of *R*_g_ to modelled daily growth (Figure 3) and no correlation to raw daily growth *ΔD*_w_ (not shown). Therefore, for further growth analyses, the results were adjusted for a 1-day time lag. *R*_g_-modelled daily growth correlation analyses yielded similar phase-to-phase characteristics for each year (Table 3). A cross-correlation analysis was also performed between *E*_S_ and modelled daily growth (i.e., without separation of respiration to maintenance and growth respiration), which showed qualitatively similar results, but revealed a bit less consistency than that of *R*_g_ to modelled daily growth (see Figure S2 available as Supplementary Data at *Tree Physiology* Online). The first phase showed no consistent *R*_g_-modelled daily growth correlation. However, a significant *r*^2^ was found in the second and third phases (except for the latter in 2011), with the second phase showing higher correlation (*P* < 0.01). On average, the second phase explained ~30% of total *R*_g_ variation and showed ~50% higher correlation than the third phase, which explained ~15% of total *R*_g_ variation. *R*_g_-raw daily growth correlation revealed inconsistencies year-to-year during the second phase compared with *R*_g_-modelled daily growth correlation. In this phase, *R*_g_-raw daily growth showed high correlation in 2011 and 2015a (*P* < 0.01), a low correlation in 2008 and 2015b, and no correlation in 2007 and 2009. The *r*^2^ significance values of *R*_g_-raw daily growth correlations in the third phase were not as consistent compared with those of *R*_g_-modelled daily growth correlations. The correlation between modelled daily growth rate and *E*_*S*_ showed very similar results to that of *R*_g_-modelled daily growth correlations for each phase and all years (see Table S1 available as Supplementary Data at *Tree Physiology* Online).

**Table 3.** Squared correlation coefficient (*r*^2^) between growth respiration (*R*_g_) and the modelled daily growth rate $\left(\Delta \hat{\Delta }G_{m}\right)$ and raw daily growth rate (*ΔD*_w_). Intra-annual growth was separated into phases that represented predominant growth processes. Phase 1 is from 1 April to the date when tracheids were first observed; Phase 2 was the period from the end of Phase 1 to a day when significant decline of growth rate occurred; and Phase 3 was the period from the end of Phase 2 to 5 October, when growth has stopped.

	Measurement	Phase 1	Phase 2	Phase 3
2007	$\Delta \hat{\Delta }G_{m}$	0.05*	0.30**	0.16**
	*ΔD*_w_	0.02	0.00	0.04
2008	$\Delta \hat{\Delta }G_{m}$	0.02	0.36**	0.15**
	*ΔD*_w_	0.08*	0.05*	0.03
2009	$\Delta \hat{\Delta }G_{m}$	0.04	0.25**	0.15**
	*ΔD*_w_	0.00	0.01	0.00
2011	$\Delta \hat{\Delta }G_{m}$	0.01	0.31**	0.01
	*ΔD*_w_	0.00	0.31**	0.04
2015a	$\Delta \hat{\Delta }G_{m}$	0.04	0.30**	0.18**
	*ΔD*_w_	0.00	0.16**	0.05
2015b	$\Delta \hat{\Delta }G_{m}$	0.01	0.22**	0.17**
	*ΔD*_w_	0.08*	0.11*	0.04

Significant levels at 0.05 and 0.01 are indicated by * and **, respectively.

### Growth efficiency

The growth efficiency (*Y*) estimated for the whole growing season varied from 0.64 to 0.81 (Table 4). Apparent (i.e., intra-annual) *Y* was higher during the second phase and declined during the third phase, although only slightly for 2015a. The highest estimated apparent *Y* during the second phase was seen in 2008, followed by 2009 and for 2015a. Meanwhile, apparent *Y* in the third phase of 2007 was lower than the other years, with 2015a showing the highest. In 2008, there was significantly more apparent biomass growth than the other years in the second phase. The apparent biomass growth was reduced considerably during the third phase, with 2008 showing the least apparent biomass growth. Apparent biomass growth in the third phase decreased more than 90% compared with their respective second phase’s apparent biomass growth except in 2015, which decreased only 59%.

**Table 4.** Growth efficiency (Y), apparent biomass growth (g (C)), annual biomass growth (g (C)) and growth respiration (g (C)) were calculated for the second (P2) and third (P3) phases. Whole year (annual) was calculated for growth efficiency and biomass growth. Both the apparent and whole year biomass growth and growth respiration values are derived variables for calculating apparent *Y* and annual *Y*, respectively. Annual *Y* and annual biomass growth include first phase values. 2015b was omitted due to insufficient data.

Year	Apparent *Y*		Annual *Y*	Apparent biomass growth		Annual biomass growth	Growth respiration	
	P2	P3		P2	P3		P2	P3
2007	0.65	0.10	0.70	89.59	6.47	179.55	47.57	67.46
2008	0.81	0.14	0.81	233.71	2.77	328.72	53.57	16.59
2009	0.78	0.31	0.74	110.59	5.42	195.89	30.91	12.12
2011	0.72	0.32	0.64	65.56	11.91	98.17	26.98	25.91
2015a	0.73	0.70	0.71	70.07	28.56	100.73	26.29	12.04
2015b	–	–	–	–	–	–	–	–

*(*P* < 0.05), **(*P* < 0.01).

### Connection between growth respiration, GPP and temperature

**Figure 4.**
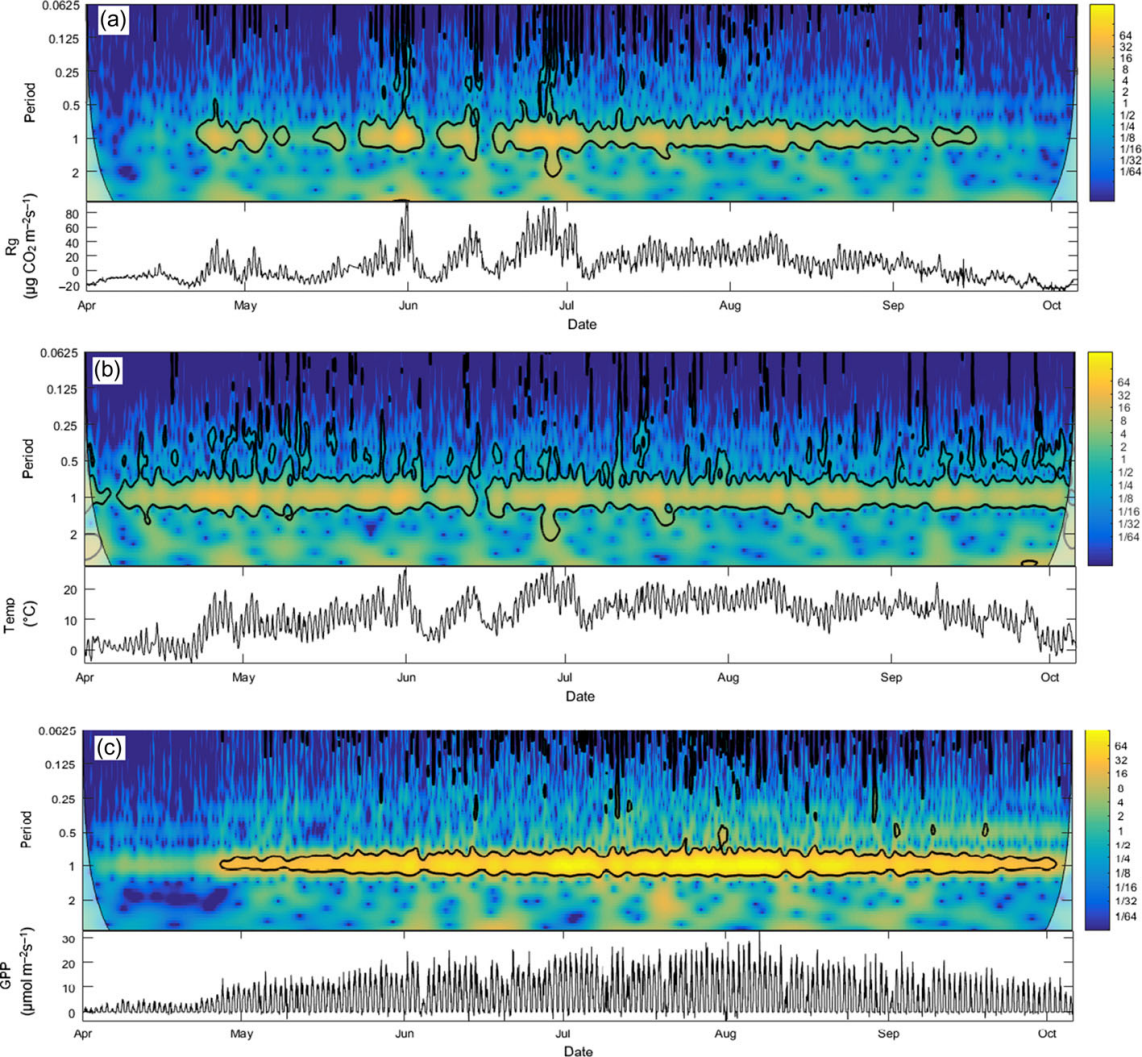
Time series (bottom) and its continuous wavelet analysis (top) of *R*_g_ (a), temperature (b) and GPP (c) in 2009 at hour–hour intervals. Within the wavelet analysis figures, the black contour lines indicate the 5% significance level and beyond black lines indicate the cone of influence where edge effects may distort the image. High and low coherence is indicated by the colours yellow and blue, respectively.

Since all years observed yielded similar results from both continuous wavelet transform (CWT) and wavelet coherence (WC) analysis, we have focused on the year 2009 in the manuscript. Figures for other years can be found in the Supplementary Data at *Tree Physiology* Online. Continuous wavelet transform of *R*_g_, GPP and temperature variables each revealed a significance at a period of 1 day, indicating a common diurnal pattern (Figure 4; see Figures S3–S6 available as Supplementary Data at *Tree Physiology* Online). From May onwards of 2009, GPP revealed a significance at the 5% level, which ended at the end of October. *R*_g_ indicated sporadic significance from May to mid-June, and from that point onwards, constant significance until early September. Meanwhile, temperature showed continual significance throughout the 2009 year.

**Figure 5.**
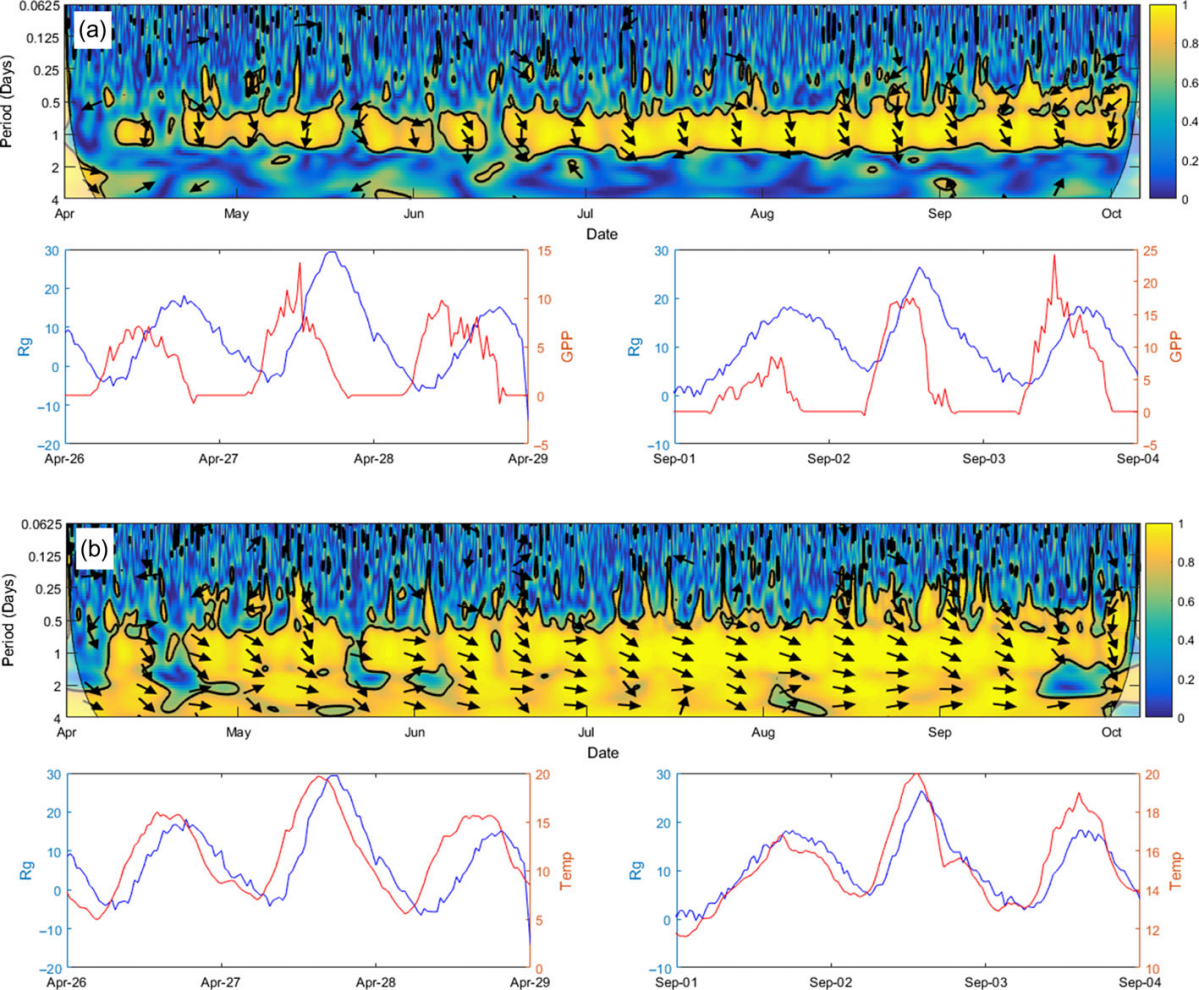
Selected time series and wavelet coherence analysis between *R*_g_ and GPP (a) and between *R*_g_ and Temperature (b) in 2009 at hour–hour intervals. The black contour lines indicate the 5% significance level and beyond black lines indicate the cone of influence where edge effects may distort the image. The phase difference (i.e., time lag) is shown by arrows (*R*_g_ lags behind). Arrows pointing right indicate no lag; down, ~6 h; and left, ~12 h. High and low coherence is indicated by the colours yellow and blue, respectively. Line figures below each WC analysis representing two, 3-day events (26–29 April; 1–4 September) show the diurnal cycle of *R*_g_ and GPP (top) and *R*_g_ and temperature (bottom). Although WC analysis is bidirectional, the line figures indicate a *R*_g_ (blue) lag to GPP and temperature (red). Refer to Table 1 for variable units.

Wavelet coherence analysis showed the strongest coherence of *R*_g_, to both GPP and temperature at a period of 1 day for all years (Figure 5; see Figures S7–S11 available as Supplementary Data at *Tree Physiology* Online). There was also evidence for sporadic significant coherence at higher frequencies (<1 day). In 2009, significant coherence was found from late April to October; coherence was also observed earlier, but it was not consistent. Coherence at higher frequencies were almost absent in *R*_g_-GPP analysis, but consistent in *R*_g_-temperature analysis. Time lags differed throughout the season. For example, *R*_g_-GPP analysis in 2009 (Figure 5a) revealed a time lag of ~6 h (*R*_g_ following GPP) in the spring, but during the remainder of the growing season, the *R*_g_-GPP lag was ~3 h. *R*_g_-temperature WC analysis however, revealed a much smaller lag (~1–2 h).

## Discussion

As shown by our results, raw dendrometer measurements used as proxy for volumetric stem growth cannot be scaled for short-term (e.g., daily) observations. This is because non-growth signals (e.g., water-related processes) are also observed from these measurements (Bäucker et al. 1998, Sevanto et al. 2011, Mencuccini et al. 2013, 2017, Chan et al. 2016, Zweifel 2016). Representing growth with the inclusion of these undesired signals may pose great challenges, as these signals generally prevail over desired signals (i.e., growth), particularly on the daily scale. By isolating these water status-related signals from raw dendrometer data and then analysing the solely growth-related signals, further in-depth investigation of growth is possible. However, our results indicate that partitioning measured *E*_S_ into functional components of growth and maintenance respiration is not wholly necessary, but may aid in complex respiration studies.

Previous studies such as Stockfors and Linder (1998) and Vose and Ryan (2002) used *ΔD*_w_ for their analyses, and to counter-act the deficiencies from using such measurements, selected dates or long intervals were used, respectively. Although direct comparison between the results from this study and previous studies may appear counter-intuitive, it draws attention to the importance of the separation of growth-related variations from water-induced changes found from dendrometer measurements.

Using the mature tissue method to separate *E*_S_ into maintenance respiration rate (*R*_m_) and growth respiration rate (*R*_g_) components revealed a relatively small and constant *R*_m_ during the growing season in comparison with growth respiration (*R*_g_) (Figure 1). To confirm the relation between growth and respiration, we conducted an additional analysis where we did not separate *E*_*S*_ into maintenance and growth components, and the results were similar to results using the mature tissue method (see Figure S1, Table S1 available as Supplementary Data at *Tree Physiology* Online).

### Link between growth respiration and stem growth

After separating intra-annual growth into phenological phases, *R*_g_ was compared with the modelled daily growth rates of $\hat{\Delta }G_{m}$$\left(\Delta \hat{\Delta }G_{m}\right)$ and raw daily growth rates (*ΔD*_w_). *R*_g_-raw daily growth correlations revealed a lack of consistency within-year and year-to-year (Table 3). This contrasted *R*_g_-modelled daily growth correlations, which showed comparable inter- and intra-annual dynamics. The dynamics of *R*_g_-modelled daily growth were similar to studies from Stockfors and Linder (1998) in *Picea abies* and Vose and Ryan (2002) in *Pinus strobus*, where they found a low correlation during the spring (first phase), increasing greatly during the summer rapid-growth period (second phase), and slightly decreasing when growth has slowed (third phase).

It is anticipated that the first phase would yield low correlation since growth was minimal during this phase. However, *R*_g_ revealed rapid, transient increases beginning 2 weeks before stem increment and a month before the onset of tracheid formation (Figure 1). Although these increases are more than half of summertime levels, the timing is prior to any tracheid development or observable radial growth, which may indicate metabolic activity due to spring reactivation. Metabolic activity is associated with cambial reactivation (e.g., cambial cell swelling) due to changes in cell biochemistry (Lavigne et al. 2004, Gruber et al. 2009). These observations agree with Lavigne et al. (2004) that the interval between high metabolic activity and xylem production may be several weeks, and may explain the *R*_g_-modelled daily growth correlation for the years 2007 and 2008 (*P* < 0.05). Here, we had microcore reference measurements of the initiation of tracheid formation but it has been reported that the new phloem cells are formed about 10–20 days before xylem cells in Scots pine (Antonova and Stasova 2006). Due to the thin phloem cell layer, death of the previous year’s phloem cells and the elasticity of living bark, associated changes in stem radius may remain masked, particularly by the hydraulic variation of the living bark in the spring. Considering this, considerable energy is used for the preparatory phases before the actual onset of tracheid formation/growth.

*R*_g_-modelled daily growth correlations in the second phase were highly significant in all years (*P* < 0.01). This is expected since this phase is characterized by a high rate of cell enlargement and growth concurrently with high photosynthetic activity (Gordon and Larson 1968). Carbohydrate concentration levels can be expected to increase due to photosynthetic activity, which ultimately promotes growth and hence respiration (Ainsworth and Bush 2011). In this phase, *R*_g_-modelled daily growth correlation had a mean *r*^2^ of 0.31, estimated with daily intervals. This result is similar to *P. strobus* (*r*^2^ = 0.32, measured with ~30 day intervals; Vose and Ryan (2002)), but slightly lower than another study with *P. sylvestris* (*r*^2^ = 0.45, measured with a mean monthly SGR; Zha et al. (2004)), and considerably lower than *P. abies* (*r*^2^ = 0.65–0.93, measured on specific dates; Stockfors and Linder (1998)). There are some factors which may explain the proportion of *R*_g_ that was not explained by stem growth. For example, growth derived from dendrometers can only observe cell division and enlargement processes but not cell wall thickening processes. This latter process may occur during the second phase and can comprise a large proportion of *R*_g_ that could not be observed from $\Delta \hat{\Delta }G_{m}$. Furthermore, CO_2_ moves axially with the xylem sap and whether it reduces or increases *E*_S_ locally largely depends on vertical location (Hölttä and Kolari 2009). We tried to minimize this effect by looking at only night-time fluxes. Additionally, non-structural carbon supply may have a large effect on stem *R*_g_, as carbon compounds are drawn from the stem reserves to areas required for growth and maintenance (Hoch et al. 2003). Although not quite relevant at this study boreal site, water stress is an environmental factor that affects respiration rates (Flexas et al. 2006). For example, respiration rates increase after a period of water stress—a necessity for photosynthetic recovery (Kirschbaum 1988).

The third phase was characterized by the decrease in cell division and cell enlargement (Deslauriers et al. 2009), and the increase in cell wall thickening processes. As a result, stem growth may not be well represented in this phase compared with the second phase, especially when using raw dendrometer measurements and modelled daily growth. Cell enlargement was observed but at a reduced level since the development of earlywood and latewood cells declined after mid-August. Generally in northern latitudes, volumetric growth reaches its maximum towards the summer solstice before declining (Leikola 1969, Rossi et al. 2006*b*). The observed decrease in *R*_g_-modelled daily growth correlation following the second phase follows observations from Vose and Ryan (2002) and Stockfors and Linder (1998). However, all years except for 2011 still showed significant *r*^2^ values. Volumetric growth in 2011 was particularly exceptional as it continued until late July before declining. The small decline in SGR below zero observed at the end of the third phase in all years may be due to the newly formed xylem conduits conducting water, which causes water to be under tension and thus, causing a decrease in stem diameter (Sperry and Sullivan 1992). Another possibility is due to decreasing osmotic strength with decreasing GPP towards autumn.

### Lagged responses between growth respiration and growth

An *R*_g_-modelled daily growth cross-correlation analysis revealed a consistent 1-day time lag of modelled daily growth preceding *R*_g_ in all years (Figure 3). This may be partly due to within-stem diffusion resistances causing stem CO_2_ efflux to lag actual stem respiration. Although not explicitly reported by Hölttä and Kolari (2009), they found that diffusion from within the stem into the atmosphere took ~2.5 h at the study site. It is therefore likely that *R*_g_ could escape from the stem to the atmosphere in less than a day. In this study, we have found *E*_S_ higher at night (22:00–05:00 h) compared with day-time values (07:00–19:00 h) at comparable temperatures (Figure 2), which may indicate two processes occurring: higher growth activity occurring at night and sap flow affecting *E*_S_ during the day. Higher growth activity affecting respiration rates bears an interesting implication on eddy covariance measurements because TER estimation is based solely on night-time values. Therefore, future GPP modelling estimations may have to take this into consideration.

Previous studies on the time lag between respiration and stem growth varied from 1 to 25 days (Zumer 1969) and from 20 to 40 days (Edwards and Hanson 1996). Other studies employed best-fit regression models (Stockfors and Linder 1998, Ceschia et al. 2002), but these have also reported similarly large differences. To contrast, some studies found a correlation with no time lag (Maier 2001, Vose and Ryan 2002, Zha et al. 2004). However, this may be due to having large intervals (e.g., monthly or bi-monthly) or averaging the SGR; which may ultimately mask the lag between respiration and SGR (Maier 2001).

### Growth efficiency

Growth efficiency (*Y*) estimated from April to October ranged from 0.64 to 0.81 (Table 4). This is within the scope of a modelling study from Roux et al. (2001) that analysed over 10 carbon-based models of tree growth, with a reported mean *Y* of 0.75. Apparent *Y* revealed similarities year-to-year except for 2015a. In the second phase, the mean apparent *Y* was ~0.74, and declined ~46% to ~0.22 in the third phase. For 2015a however, apparent *Y* in the third phase was as high as its second phase. This is because the SGR did not decline as suddenly as in the other years (Figure 1). Generally, the large contrast between these two phases may be due to differences in the allocation of carbon. Much of the carbon in the second phase was apportioned to volumetric increase (e.g., cell enlargement). This observation would further support the high *R*_g_-modelled daily growth correlation observed during this phase. In the third phase, apparent *Y* estimates showed an apparent decline in new structural development. However, it is clear that *R*_g_ remains rather high (Figure 1). Therefore, the proportion of *R*_g_ not explained by apparent *Y* and *R*_g_-modelled daily growth dynamics may be due to secondary thickening and deposition of new materials (e.g., cellulose, lignin and cutin) (Gričar et al. 2005, Rossi et al. 2006*a*) and not detectable by radial stem measurements.

### Link between *R*_g_ and both GPP and temperature

Since all variables analysed with the wavelet analysis exhibited similar patterns, the interpretation of the analysis is not straightforward. Unlike the *r*^2^ analyses in this study, the current wavelet analysis requires day-time values of *R*_g_ to conduct the analysis. While night-time values were uniquely used in the other analyses of this study, day values were used for the wavelet analysis as continuous data is required for it. Therefore, this analysis should be taken with caution as the transport of CO_2_ with the xylem sap has not been taken into account. The WC analysis between *R*_g_ and other variables showed a significant coherence at the period of one day, demonstrating a common daily cycle and tight correlation between these measured variables. However, it is important to remember that this does not necessarily indicate causality, although it would be tempting to conclude that variations in GPP and recently photosynthetically fixed carbohydrates were causing variation in *R*_g_ (Figure 5); as has been shown, e.g., in Zha et al. (2004). The *R*_g_-GPP time lag of ~3 h corresponds to the time scale of sugar concentration propagation rate in the phloem between the foliage and the stem (Mencuccini et al. 2013), and the *R*_g_-temperature time lag of ~2 h corresponds to the time scale of the radial diffusion of respired CO_2_ out from the stem. From the same study site for the same species, the *R*_g_-temperature time lag is similar to the result from (Hölttä and Kolari 2009). In mid-April, coherence was found between both GPP and temperature with *R*_g_. This finding correlates well with the results found from *R*_g_-modelled daily growth correlations during the first phase, which may indicate metabolic activity due to spring reactivation.

## Conclusions

Our study has demonstrated that dendrometers are powerful instruments to study the stem growth of trees provided that the reversible daily variations in stem water status are accounted for. By using dendrometer measurements in conjunction with other physiological measurements, we are able to link whole-tree physiological processes. In this study, a significant correlation was found between stem growth and stem CO_2_ efflux. As a first, we were able to study the relationships between stem growth, GPP and growth respiration on a daily time scale over the course of the growing season and over several years. This analysis revealed two important physiological observations: that the relationship between *R*_g_ and growth differed substantially at different times of the year, and that there was a consistent 1-day time lag of *R*_g_ to growth. This study demonstrates the need for further in-depth analysis of growth and respiration dynamics—especially pertaining to cellular respiration at specific developmental stages, its woody tissue costs and linkages to source–sink processes.

## Supplementary Material

Supplementary Figure 1

Supplementary Figure 2

Supplementary Figure 3

Supplementary Figure 4

Supplementary Figure 5

Supplementary Figure 6

Supplementary Figure 7

Supplementary Figure 8

Supplementary Figure 9

Supplementary Figure 10

Supplementary Figure 11

Supplementary Table 1

Supplementary Data
