# Supplementary figures and images for: Linking stem growth respiration to the seasonal course of stem growth and GPP of Scots pine
Source: Tree Physiol. 2018 May 16;38(9):1356–70. doi: 10.1093/treephys/tpy040 (PMC6178967; doi:10.1093/treephys/tpy040)

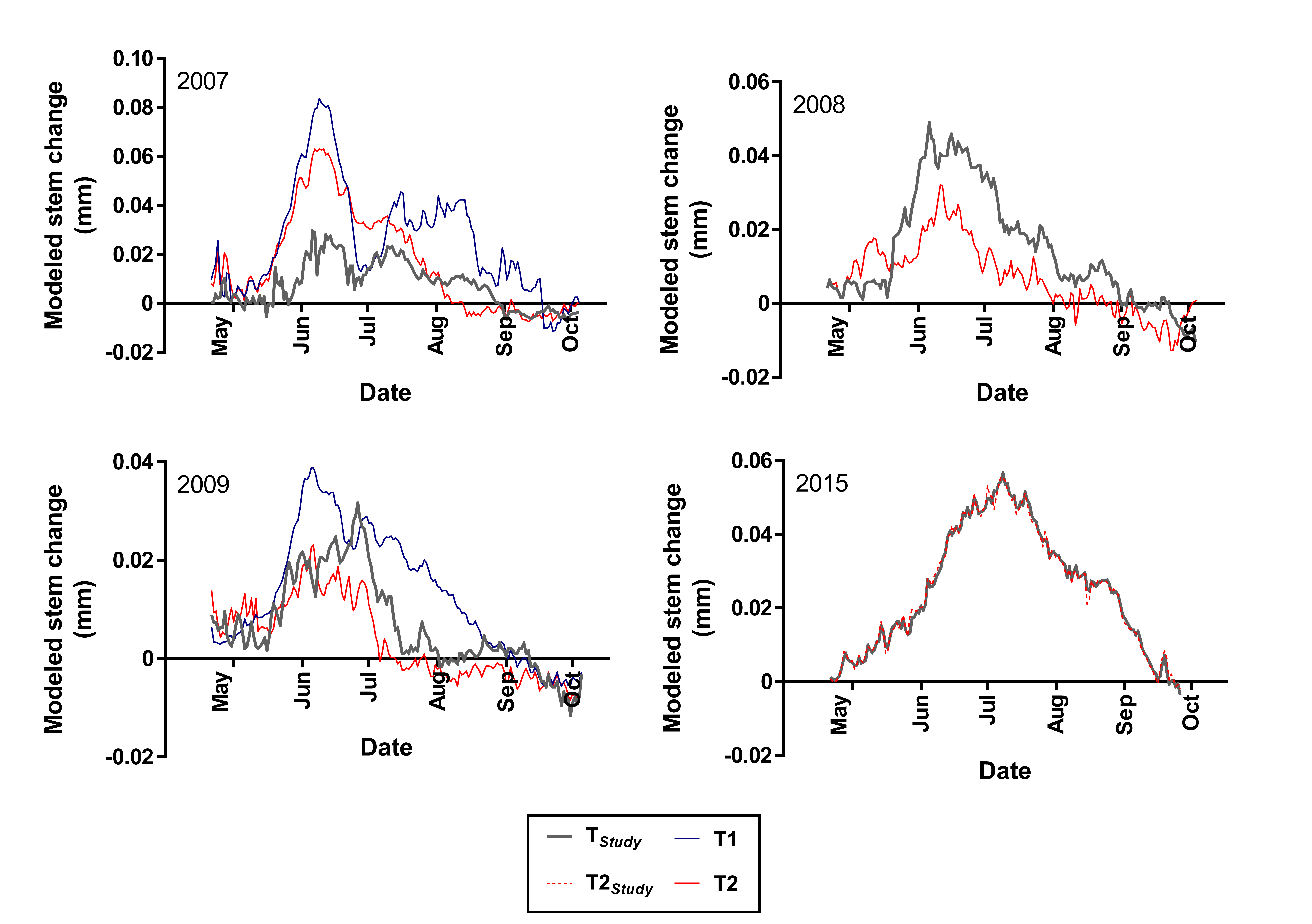

Supplement: Supplementary Figure 1 [file tpy040figures1.png]

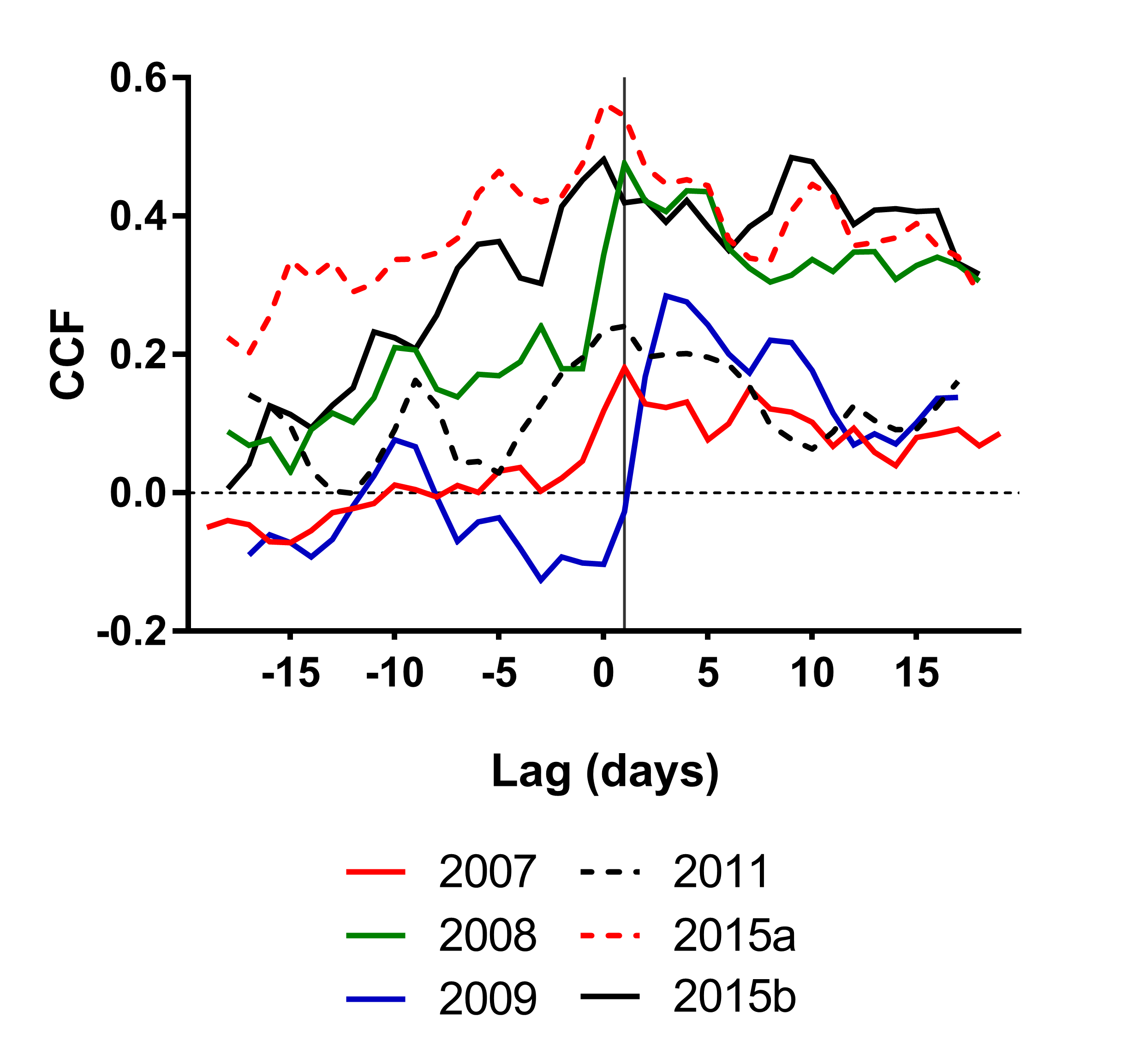

Supplement: Supplementary Figure 2 [file tpy040figures2.png]

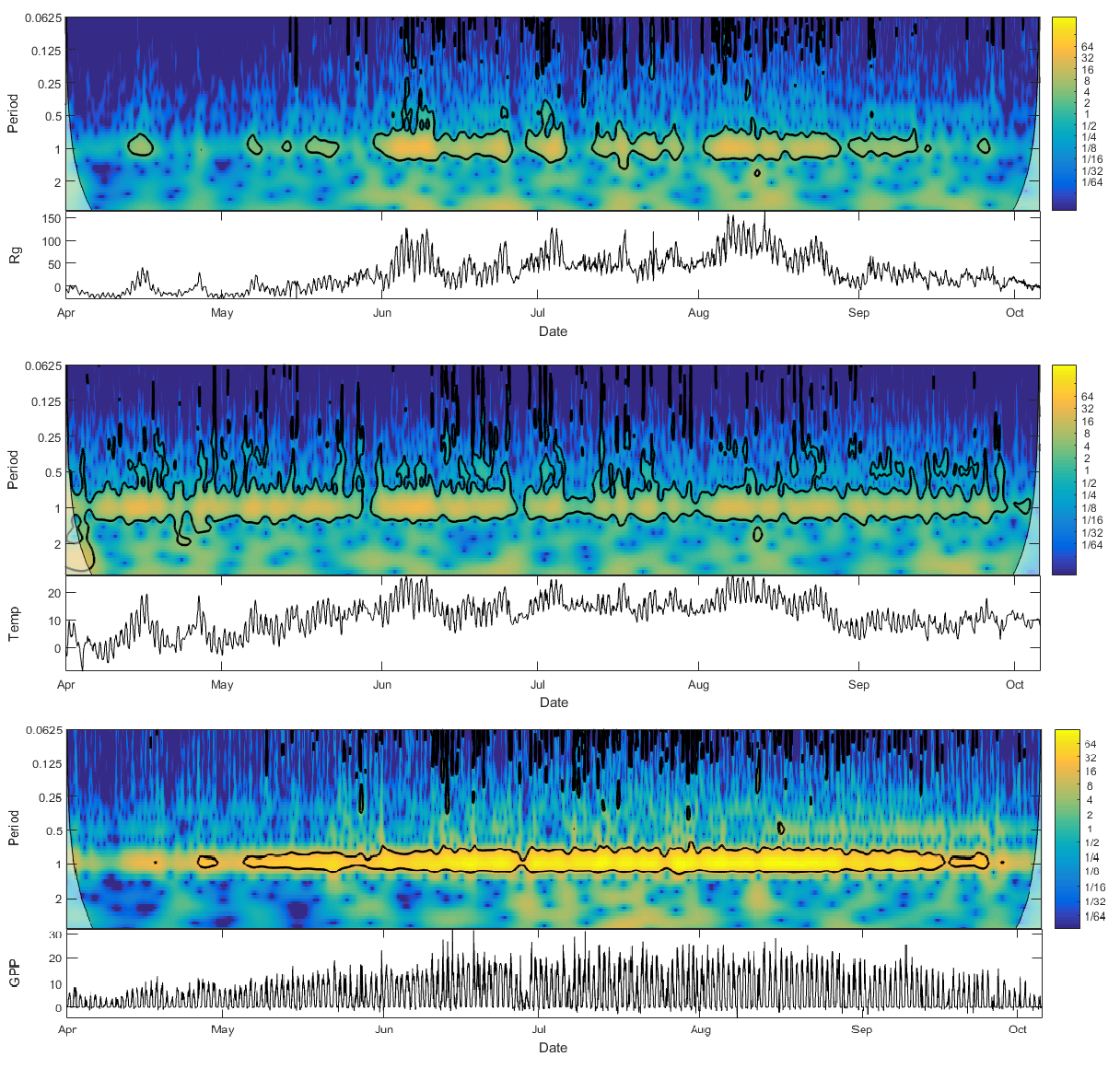

Supplement: Supplementary Figure 3 [file tpy040figures3.png]

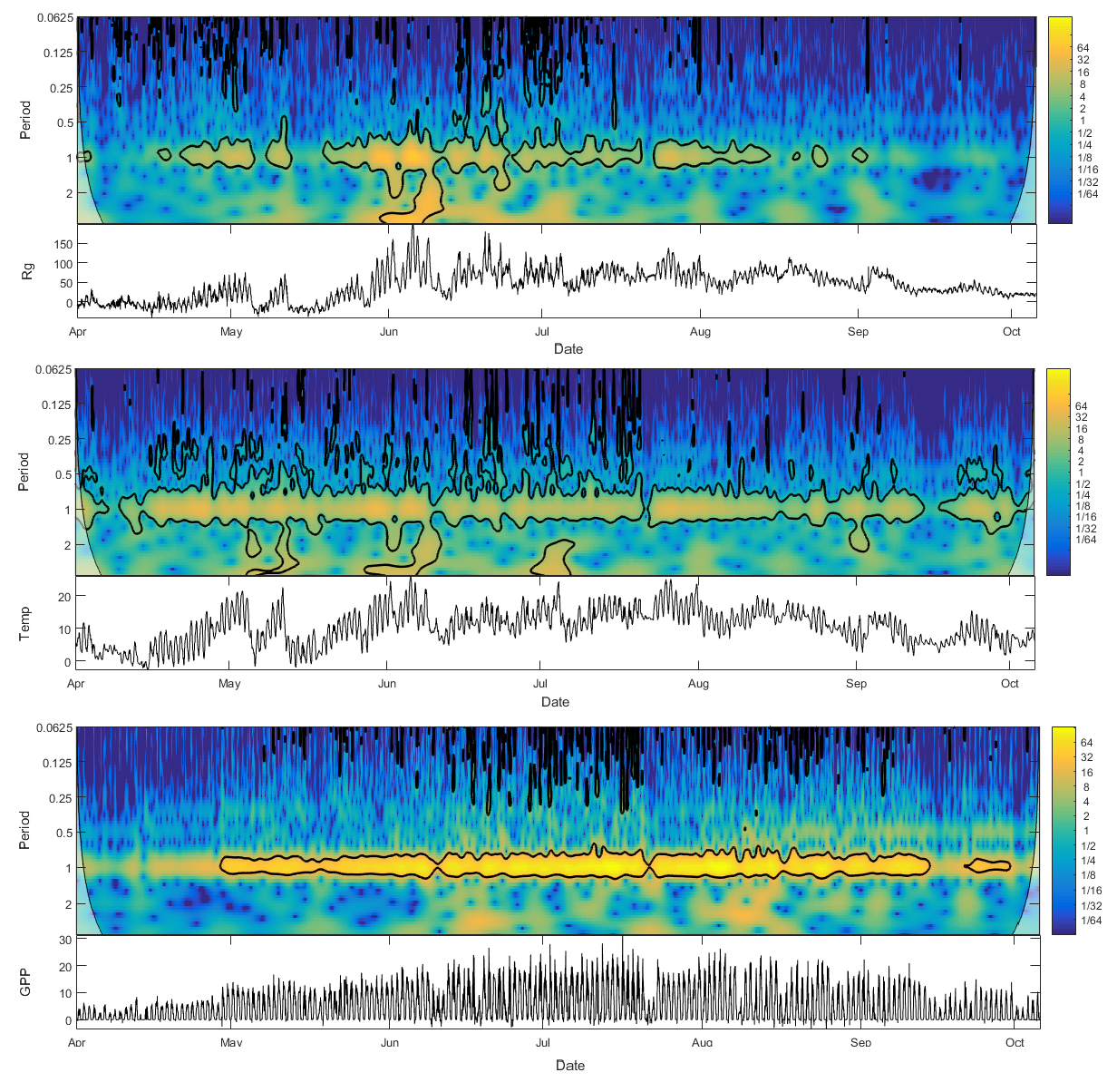

Supplement: Supplementary Figure 4 [file tpy040figures4.png]

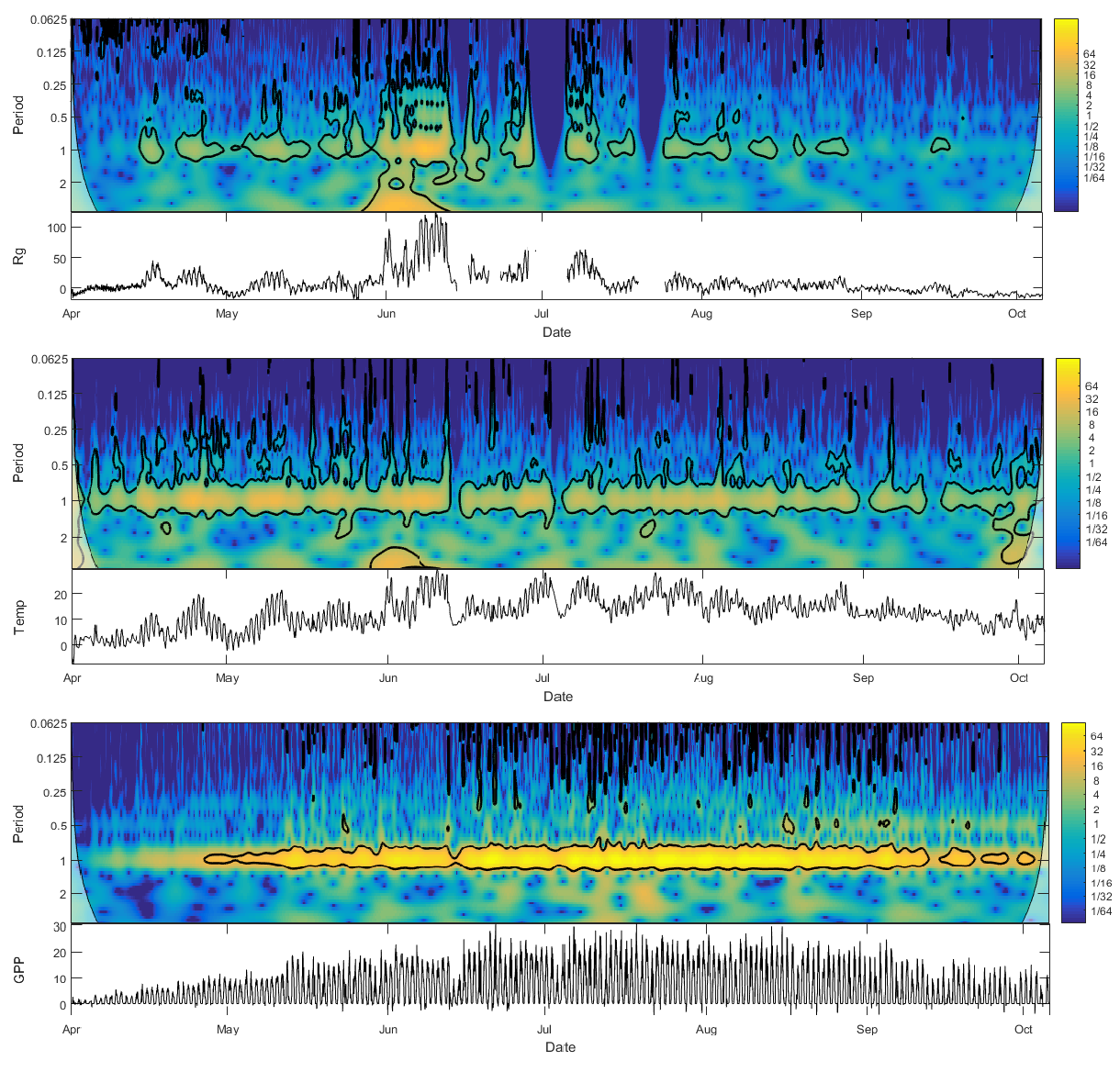

Supplement: Supplementary Figure 5 [file tpy040figures5.png]

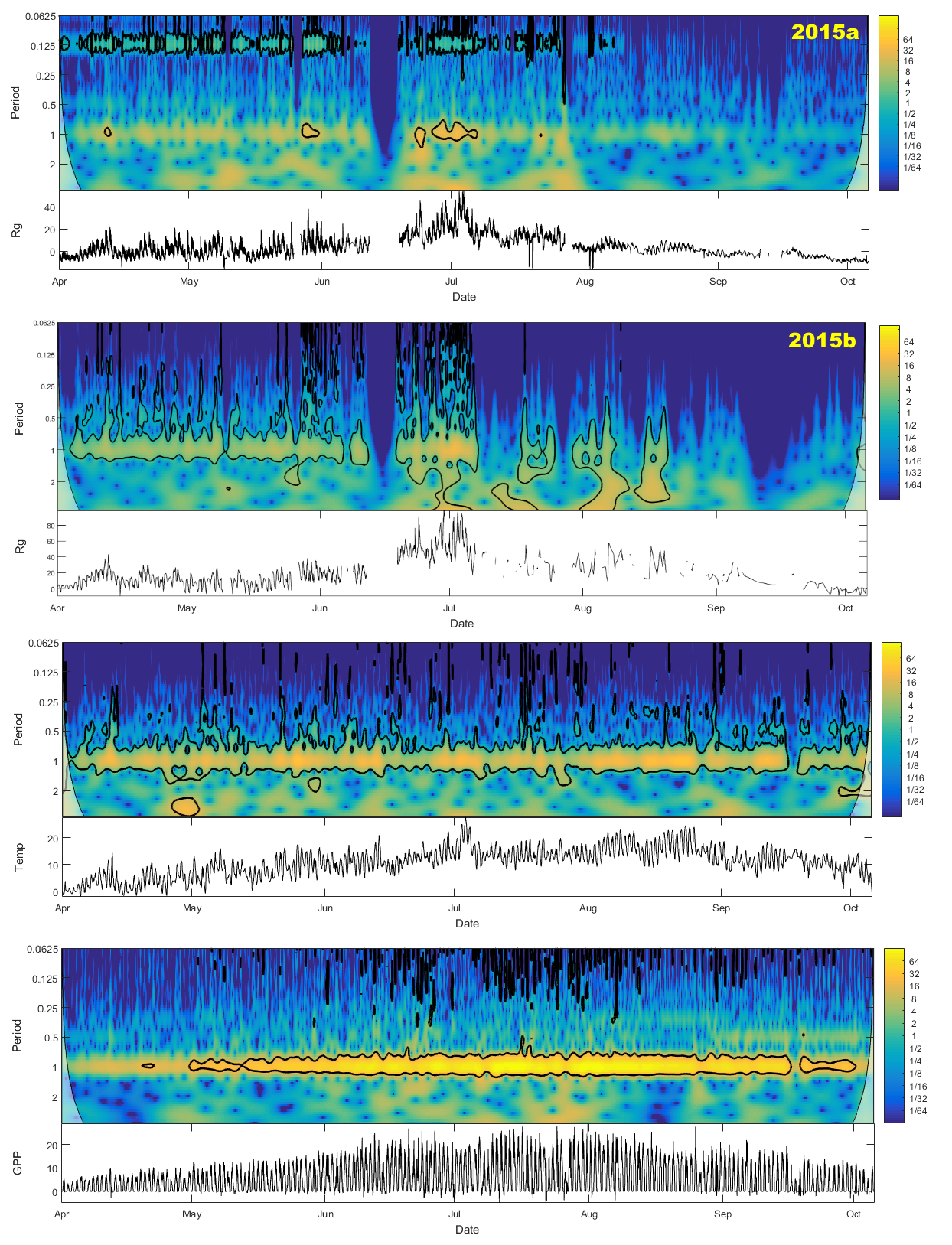

Supplement: Supplementary Figure 6 [file tpy040figures6.png]

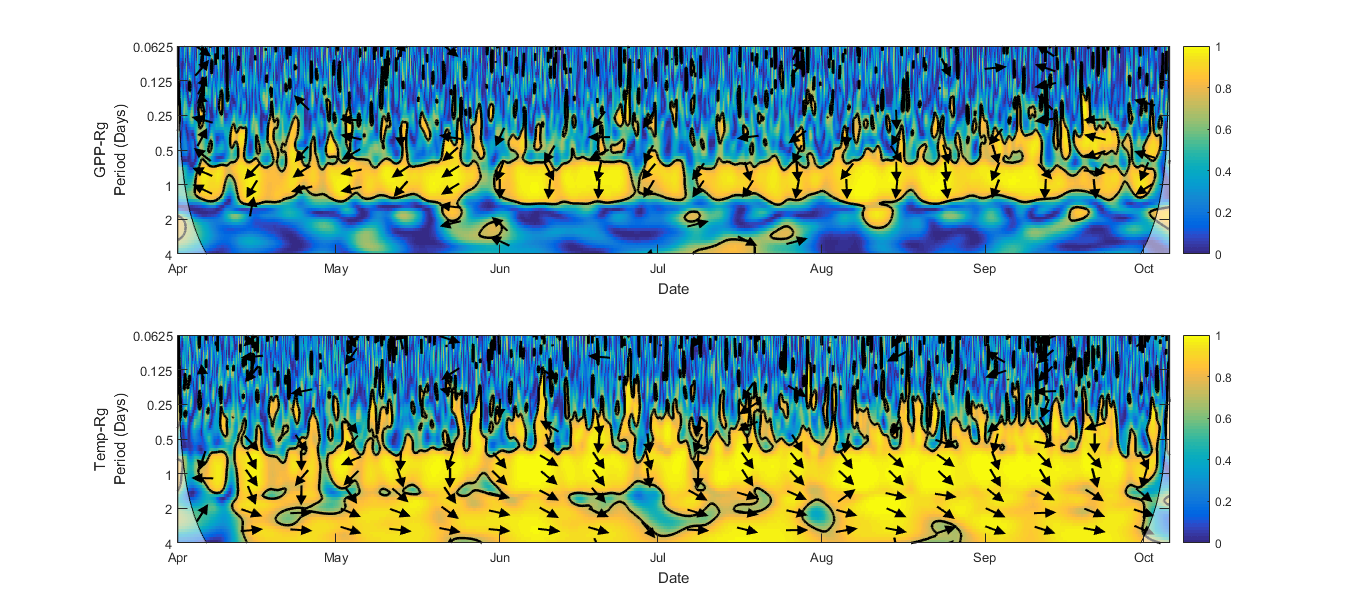

Supplement: Supplementary Figure 7 [file tpy040figures7.png]

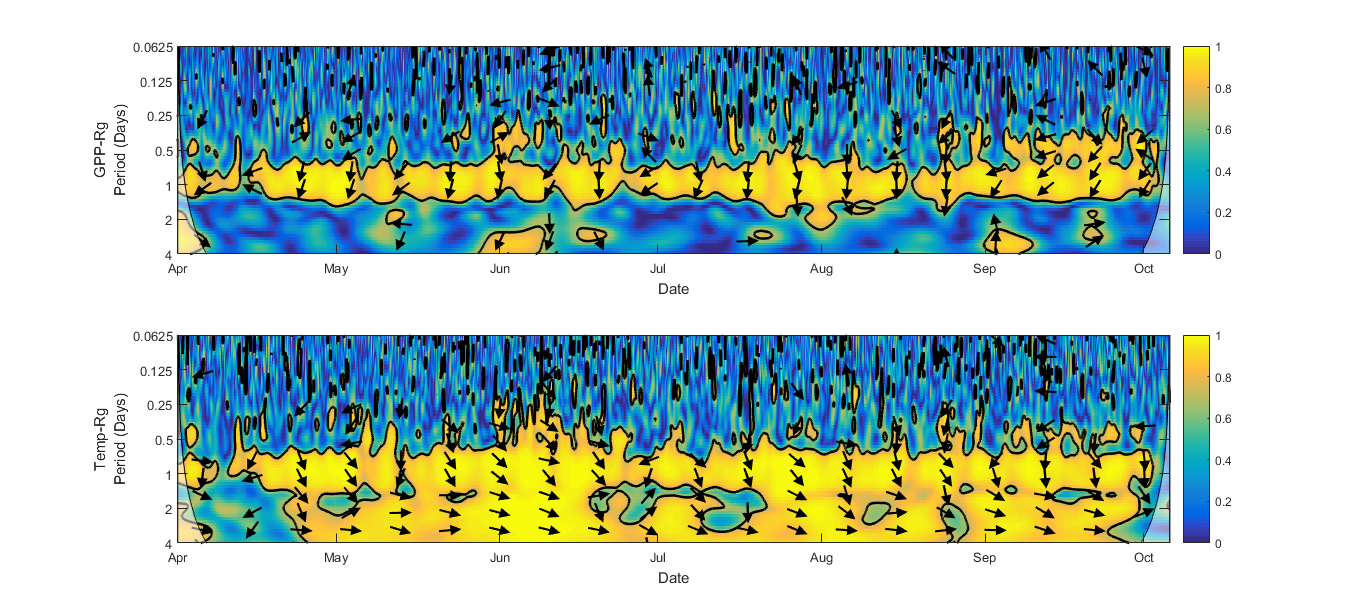

Supplement: Supplementary Figure 8 [file tpy040figures8.png]

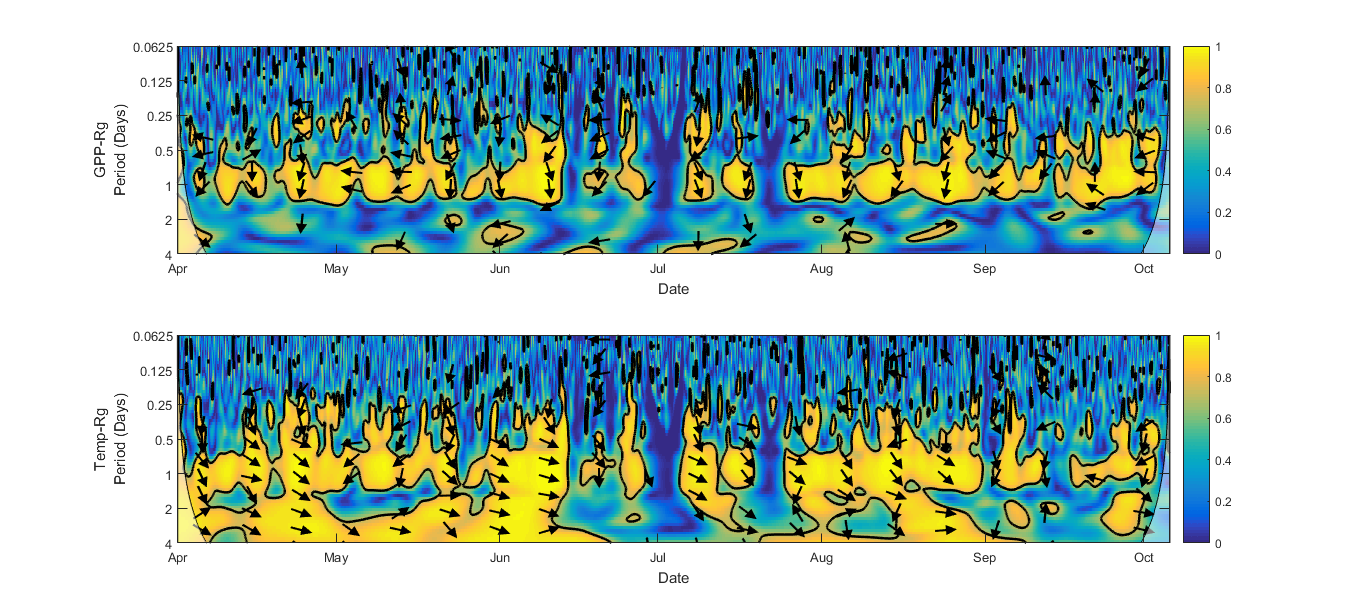

Supplement: Supplementary Figure 9 [file tpy040figures9.png]

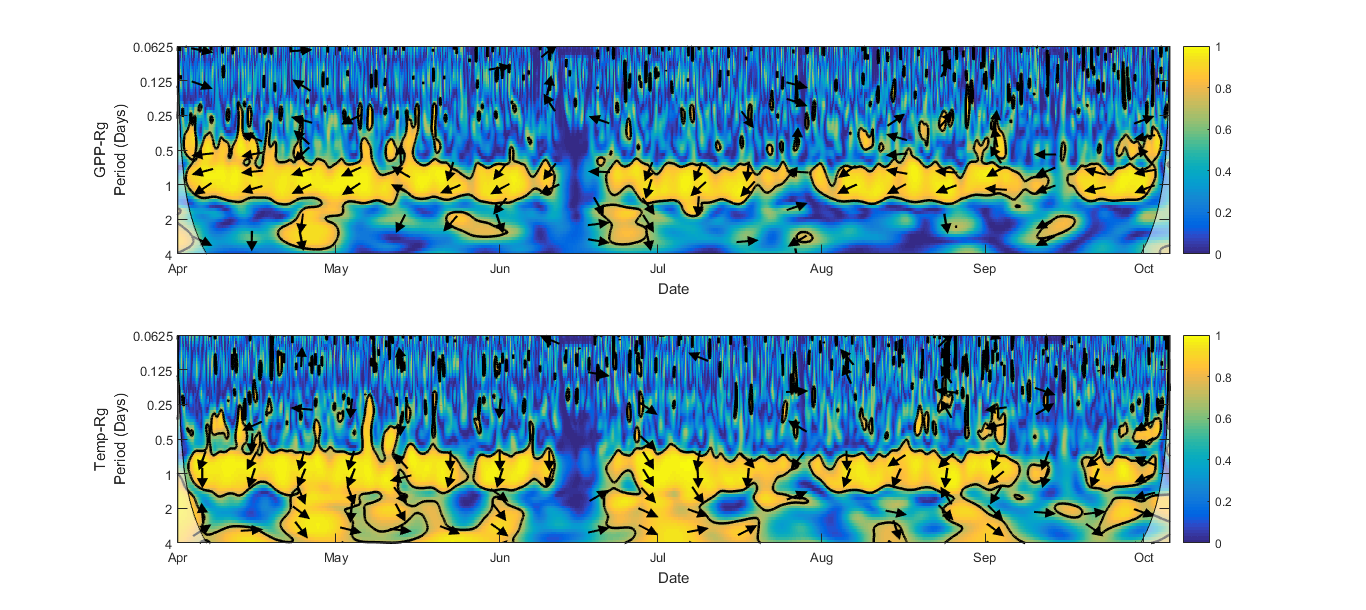

Supplement: Supplementary Figure 10 [file tpy040figures10.png]

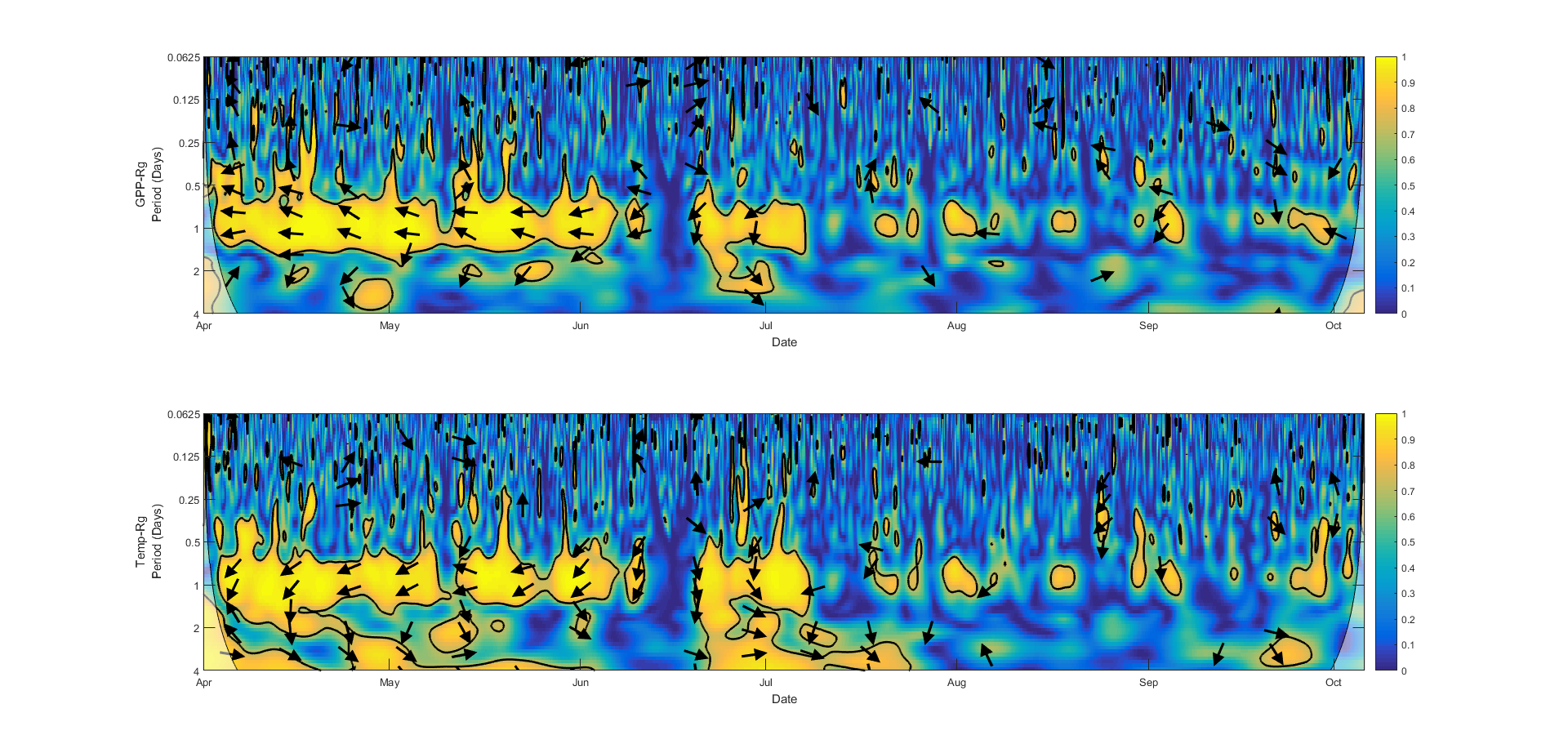

Supplement: Supplementary Figure 11 [file tpy040figures11.png]
