# Supplementary material for: Linking stem growth respiration to the seasonal course of stem growth and GPP of Scots pine
Source: Tree Physiol. 2018 May 16;38(9):1356–70. doi: 10.1093/treephys/tpy040 (PMC6178967; doi:10.1093/treephys/tpy040)
Supplement: Supplementary Table 1 [file tpy040tables1.pdf]

Table S1. Squared correlation coefficient (r^2) between stem CO_2 efflux (E_S) and the estimated growth rate ($\Delta\hat{\Delta}G_m$). Intra-annual growth was separated into phases that represented predominant growth processes. Phase 1 is from April 1 to the date when tracheids were first observed; Phase 2 was the period from the end of Phase 1 to a day when significant decline of growth rate occurred: and Phase 3 was the period from the end of Phase 2 to October 5, when growth has stopped.

* ($P < 0.05$), ** ($P < 0.01$)

	Measurement	Phase 1	Phase 2	Phase 3
2007	$\Delta\hat{\Delta}G_m$	0.05*	0.27**	0.16**
2008	$\Delta\hat{\Delta}G_m$	0.02	0.36**	0.16**
2009	$\Delta\hat{\Delta}G_m$	0.05*	0.11**	0.09**
2011	$\Delta\hat{\Delta}G_m$	0.00	0.31**	0.00
2015a	$\Delta\hat{\Delta}G_m$	0.04	0.26**	0.20**
2015b	$\Delta\hat{\Delta}G_m$	0.02	0.23**	0.20**
