# Supplementary material for: Linking stem growth respiration to the seasonal course of stem growth and GPP of Scots pine
Source: Tree Physiol. 2018 May 16;38(9):1356–70. doi: 10.1093/treephys/tpy040 (PMC6178967; doi:10.1093/treephys/tpy040)
Supplement: Supplementary Data [file tpy040supportinginformation.docx]

**Supporting Information**

**
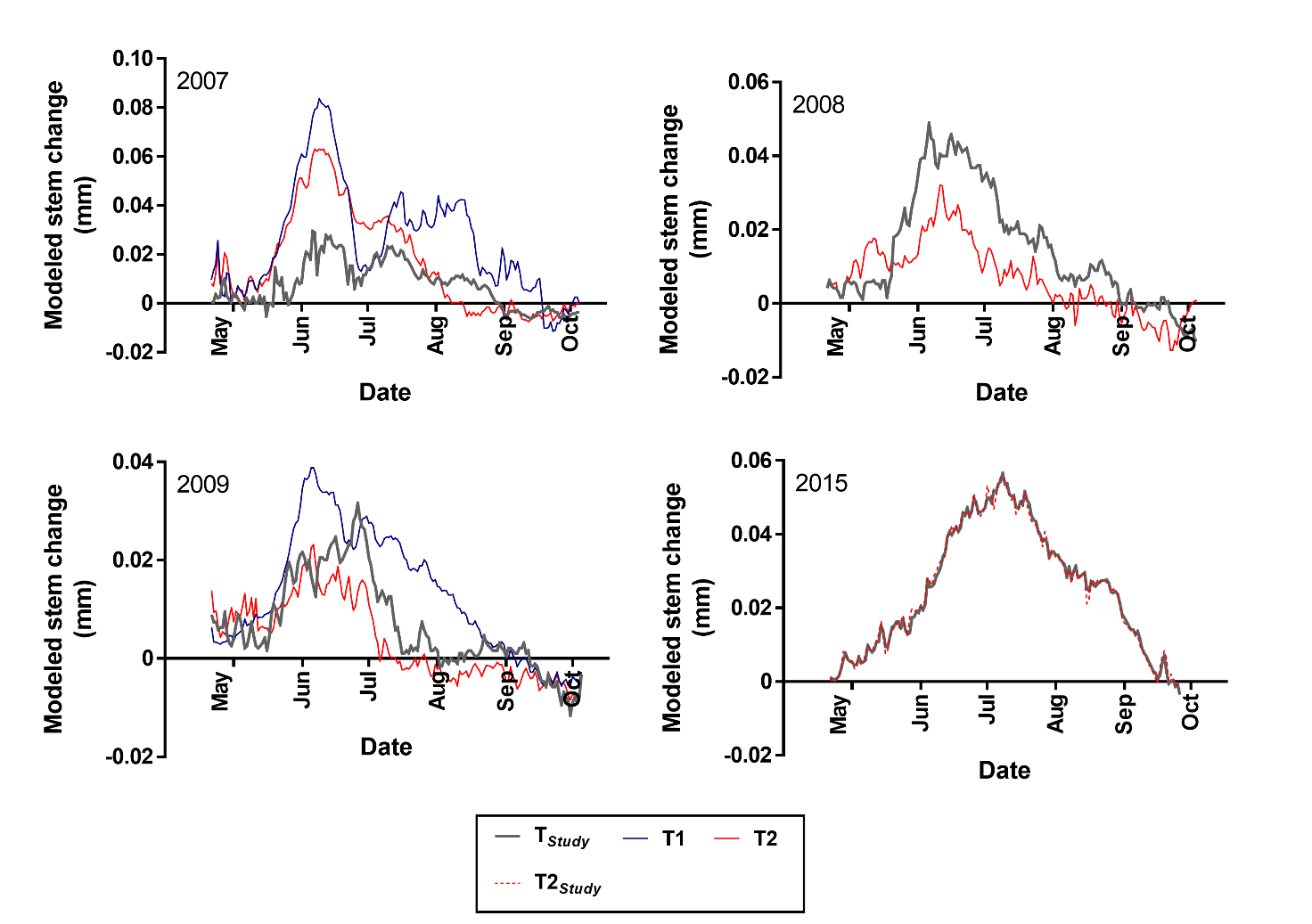
**

Fig. S1. Calculated daily derivatives of modelled daily growth rate ($\Delta\hat{\Delta}G_{m}$), averaged over 10 days for visualization. T*_Study_* (dark grey) denotes the tree used in the study, while T2*_Study_* (dotted red) denotes the second tree used in 2015. T1 (blue) and T2 (red) denote the respective trees that measured radial stem variations only (i.e. stem CO_2_ efflux was not measured on these trees). Note that all correlation analyses in this study used daily (non-averaged) values.


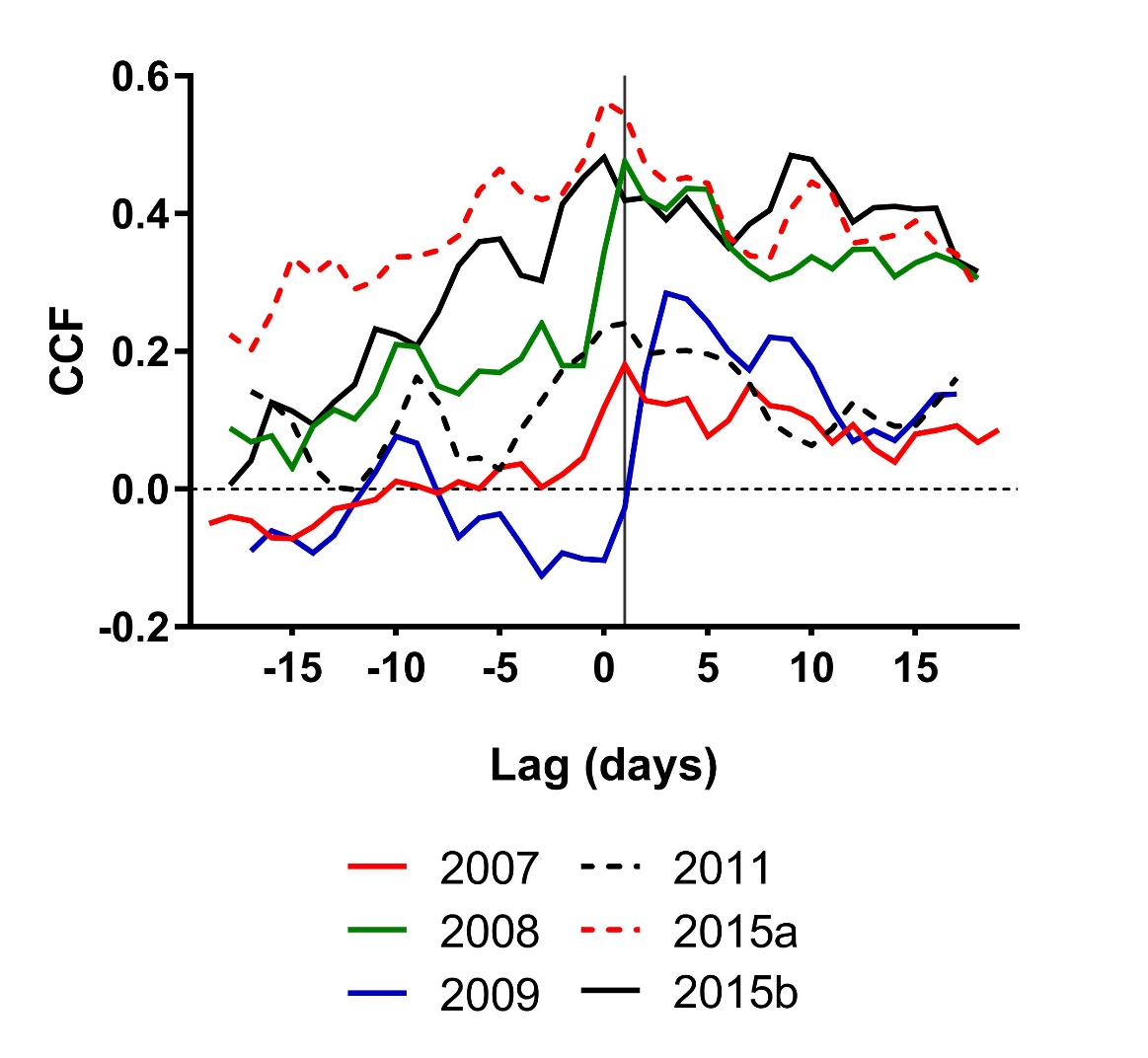


Fig. S2. Cross-correlation function of daily-averaged, night-time values of stem CO_2_ efflux (*E_S_*) to modelled daily growth rate ($\Delta\hat{\Delta}G_{m}$) for all years studied. Dotted horizontal line indicates zero point. Vertical solid line indicates period with highest correlation.


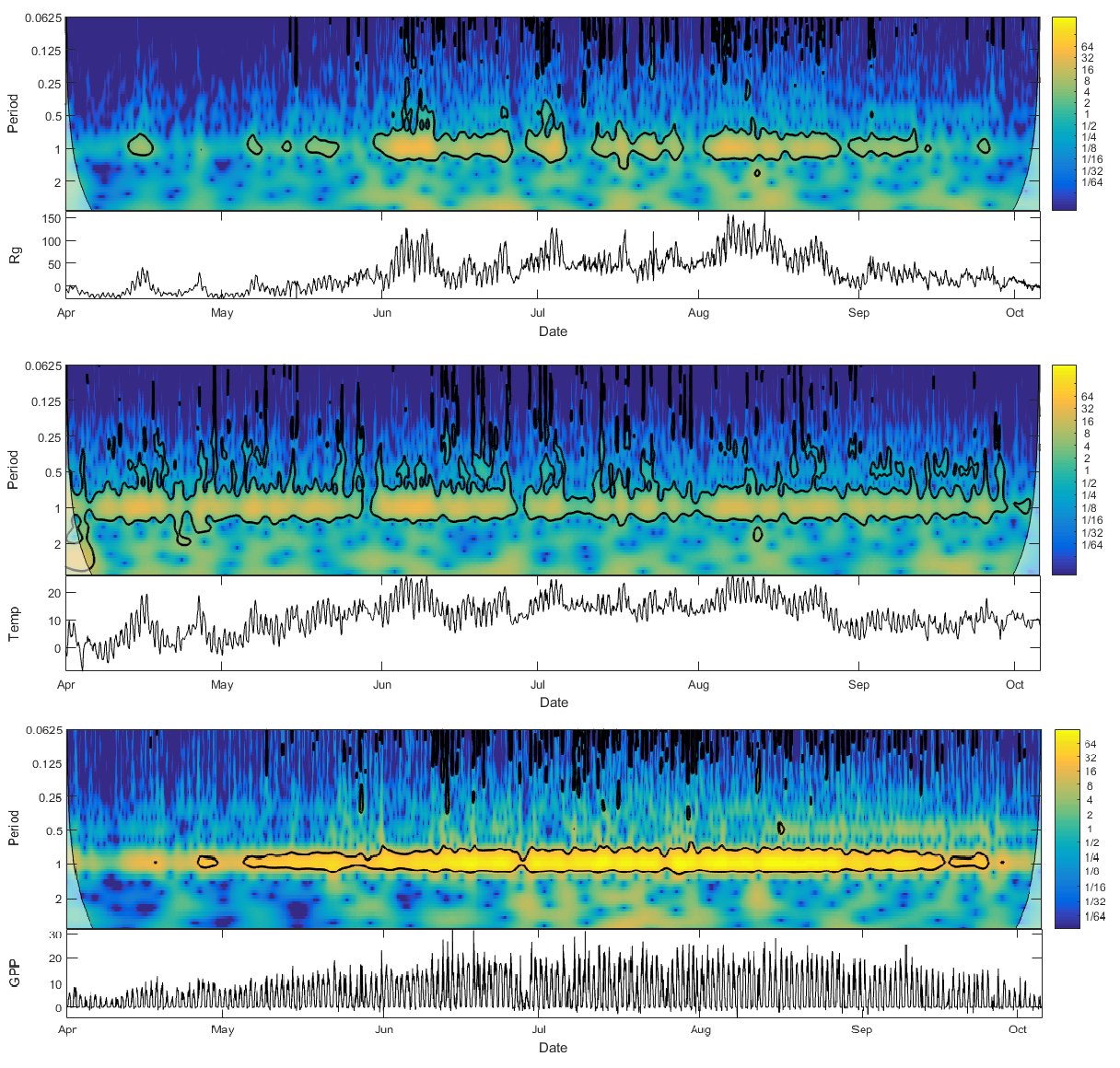


Fig. S3. Time series and its continuous wavelet analysis of *R*_g_ (top), temperature (middle) and GPP (bottom) in 2007. Within the wavelet analysis figures, the black contour lines indicate the 5% significance level and beyond black lines indicate the cone of influence where edge effects may distort the image. Refer to Table 1 for variable units.


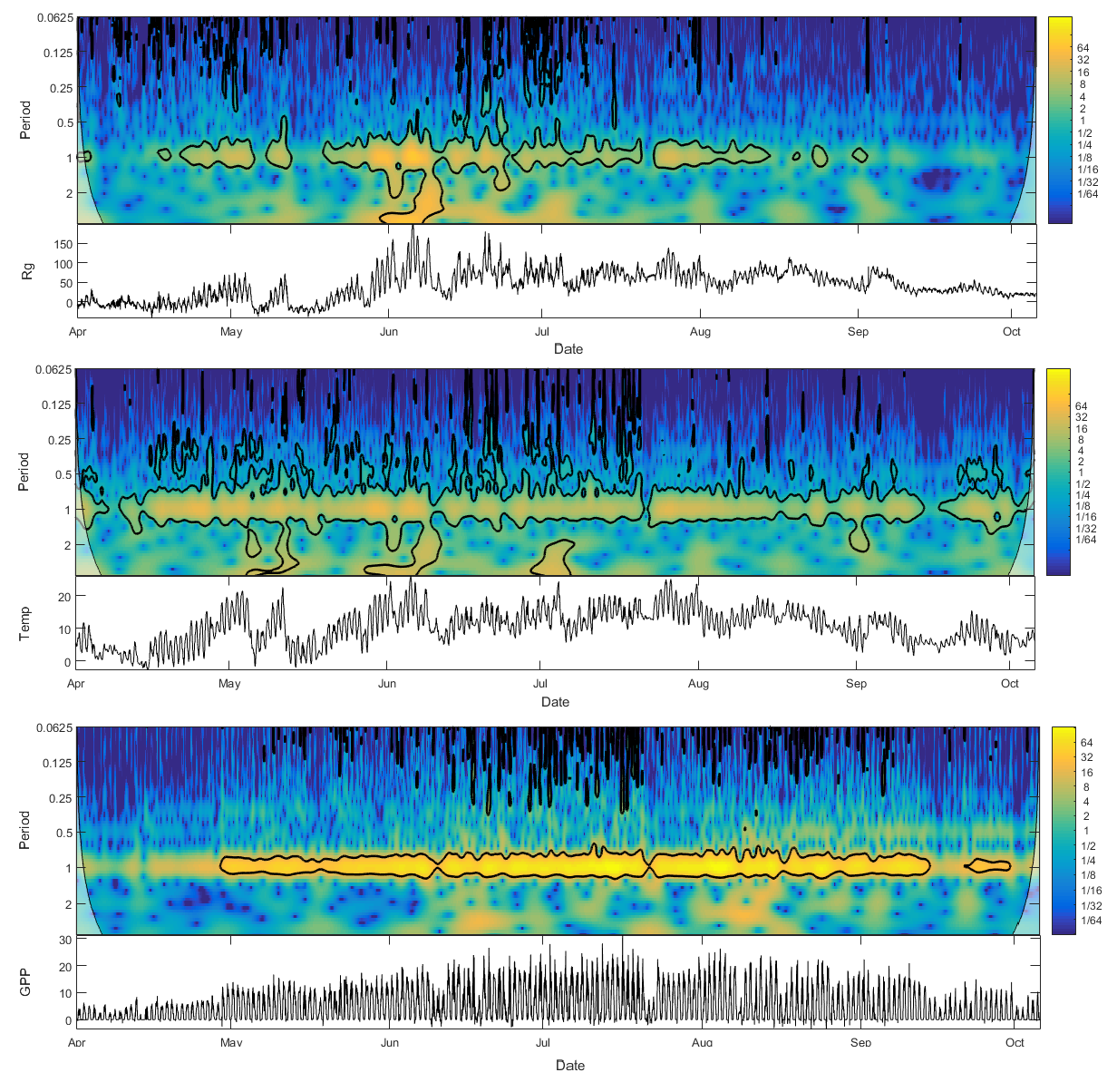


Fig. S4. Time series and its continuous wavelet analysis of *R*_g_ (top), temperature (middle) and GPP (bottom) in 2008. Within the wavelet analysis figures, the black contour lines indicate the 5% significance level and beyond black lines indicate the cone of influence where edge effects may distort the image. Refer to Table 1 for variable units.


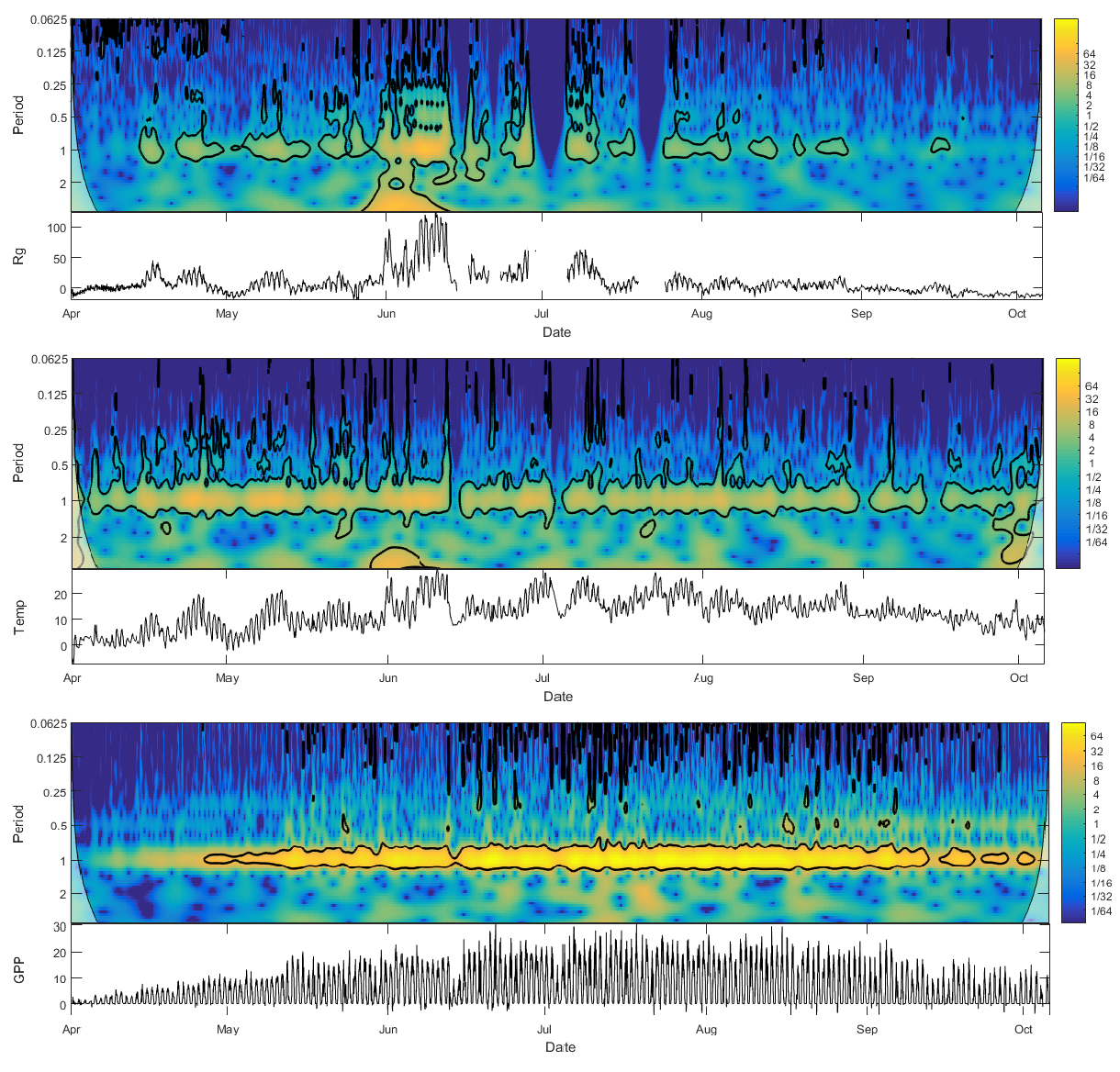


Fig. S5. Time series and its continuous wavelet analysis of *R*_g_ (top), temperature (middle) and GPP (bottom) in 2011. Within the wavelet analysis figures, the black contour lines indicate the 5% significance level and beyond black lines indicate the cone of influence where edge effects may distort the image. Refer to Table 1 for variable units.


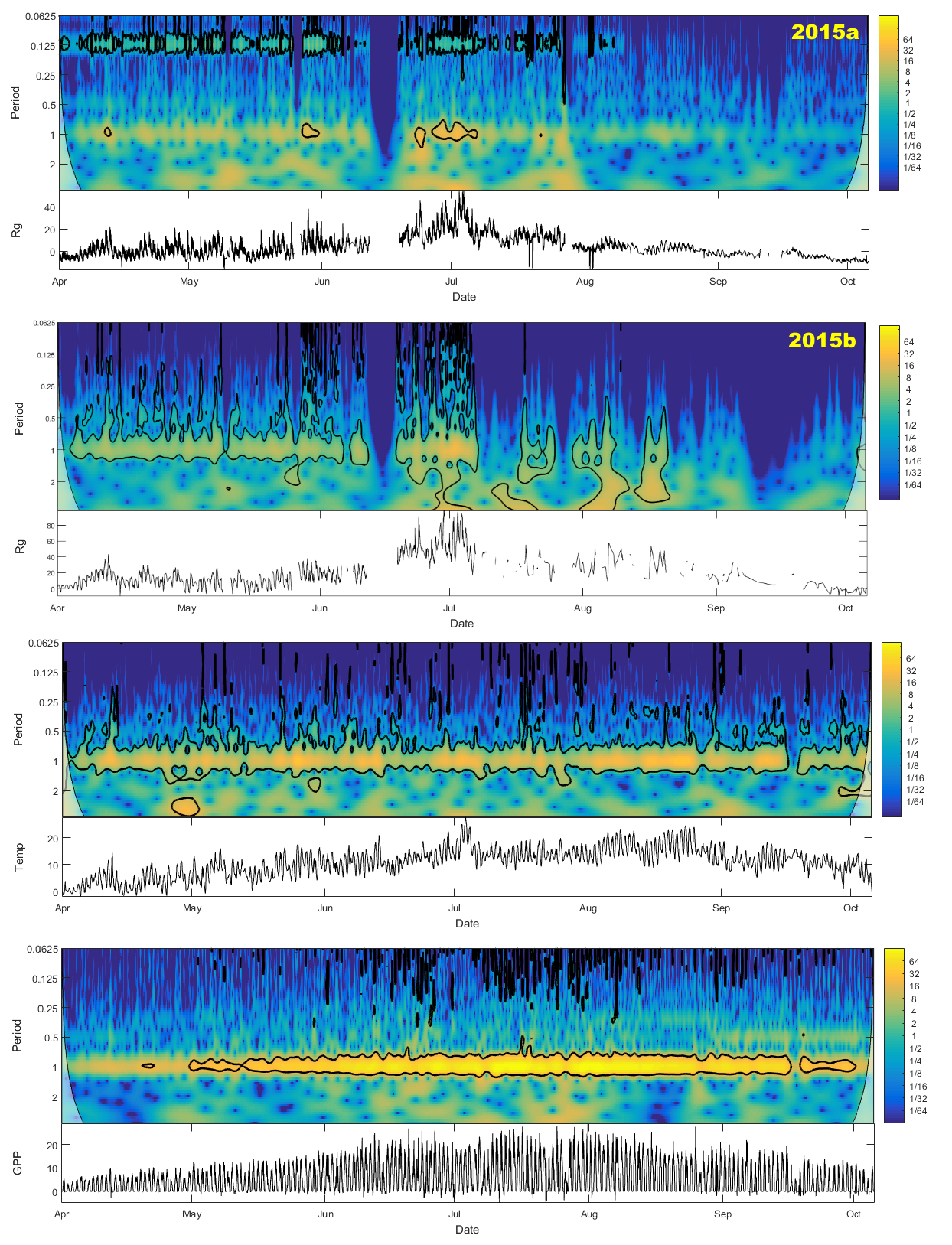


Fig. S6. Time series and its continuous wavelet analysis of *R*_g_ (top), temperature (middle) and GPP (bottom) in 2015. 2015a and 2015b denote tree #1 and #2, respectively. Within the wavelet analysis figures, the black contour lines indicate the 5% significance level and beyond black lines indicate the cone of influence where edge effects may distort the image. Refer to Table 1 for variable units.


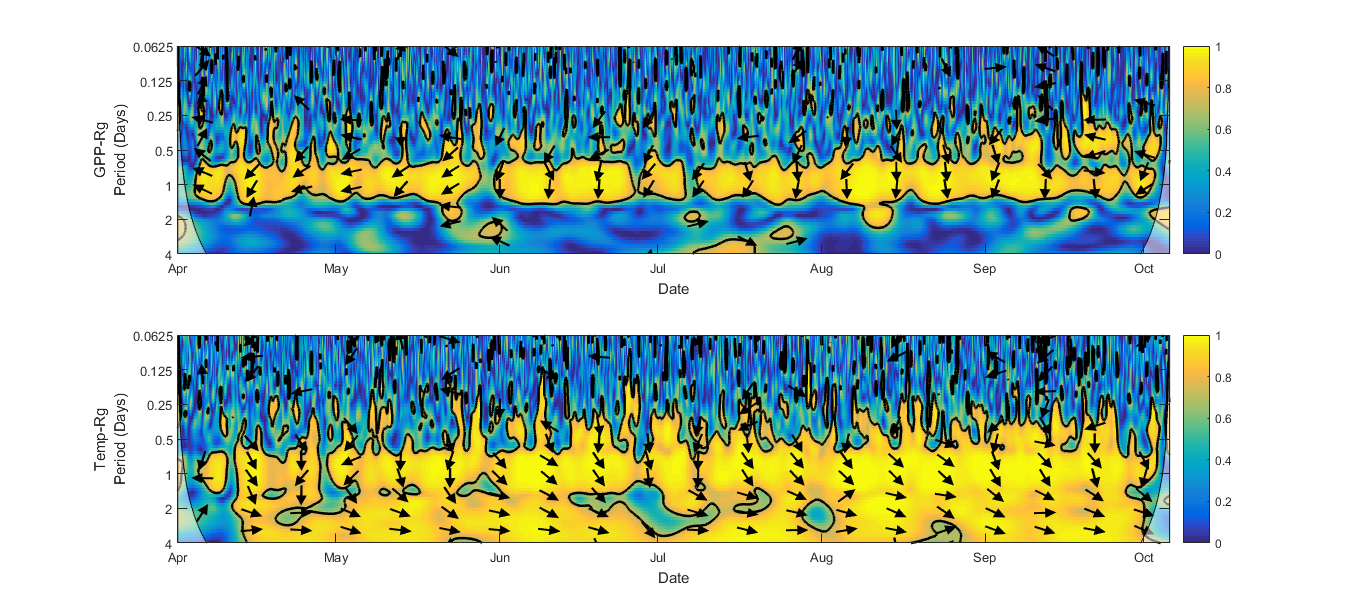


Fig. S7. Wavelet coherence analysis between *R*_g_ and GPP (top) and *R*_g_ and temperature (bottom) in 2007. The black contour lines indicate the 5% significance level and beyond black lines indicate the cone of influence where edge effects may distort the image. The phase difference (i.e. time lag) is shown by arrows (*R*_g_ lags behind). Arrows pointing right indicate no lag; down, ~6 h; and left, ~12 h. Refer to Table 1 for variable units.


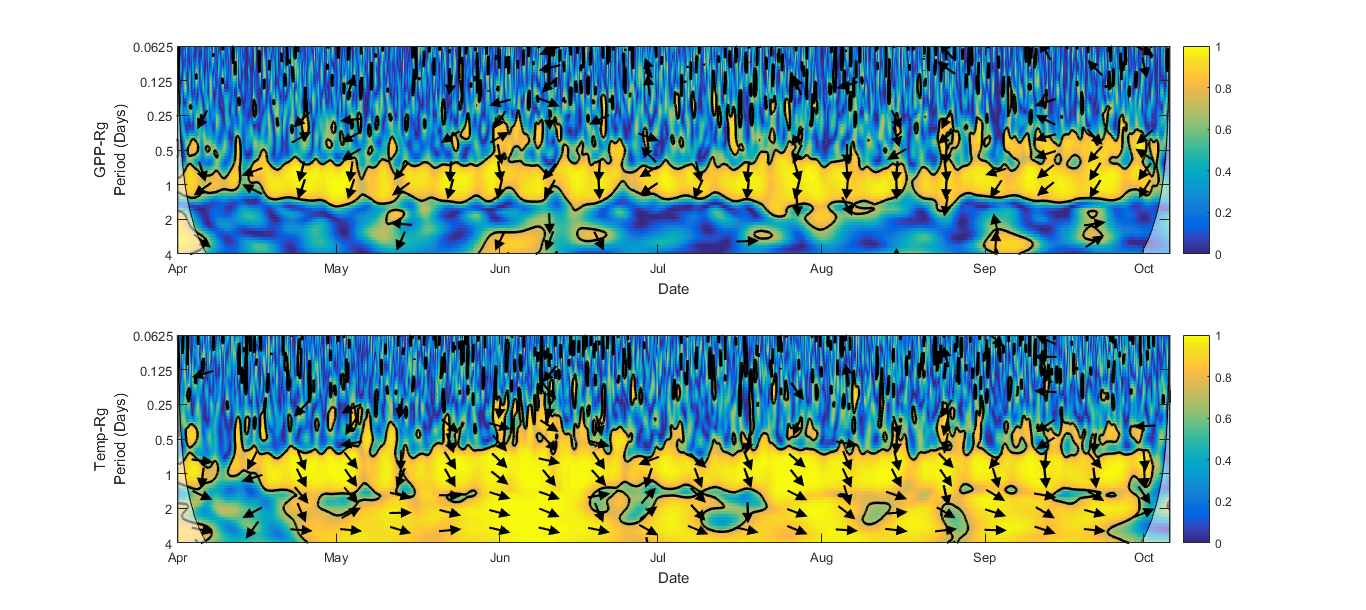


Fig. S8. Wavelet coherence analysis between *R*_g_ and GPP (top) and *R*_g_ and temperature (bottom) in 2008. The black contour lines indicate the 5% significance level and beyond black lines indicate the cone of influence where edge effects may distort the image. The phase difference (i.e. time lag) is shown by arrows (*R*_g_ lags behind). Arrows pointing right indicate no lag; down, ~6 h; and left, ~12 h. Refer to Table 1 for variable units.


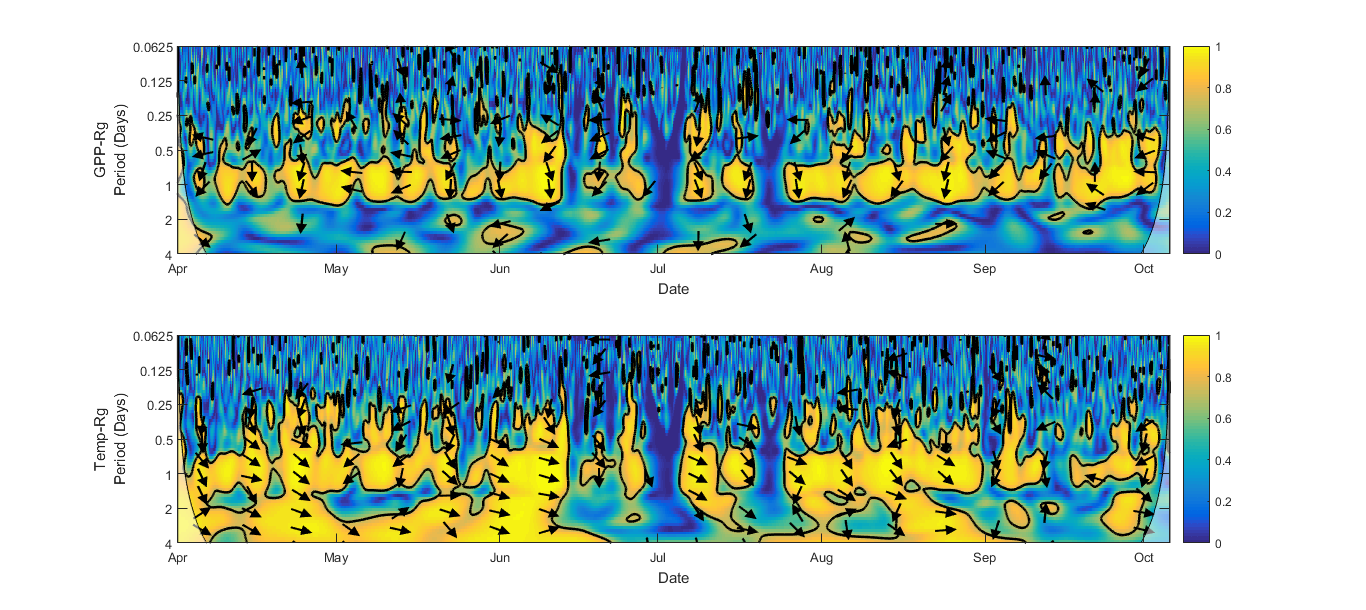


Fig. S9. Wavelet coherence analysis between *R*_g_ and GPP (top) and *R*_g_ and temperature (bottom) in 2011. The black contour lines indicate the 5% significance level and beyond black lines indicate the cone of influence where edge effects may distort the image. The phase difference (i.e. time lag) is shown by arrows (*R*_g_ lags behind). Arrows pointing right indicate no lag; down, ~6 h; and left, ~12 h. Refer to Table 1 for variable units.


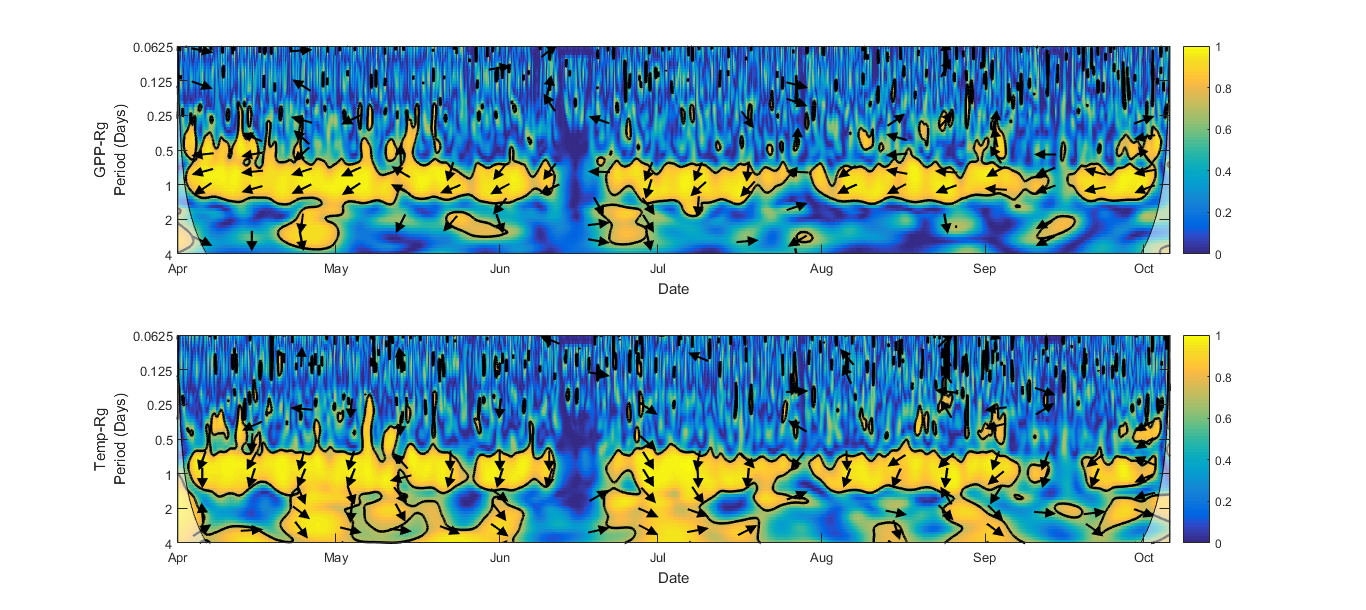


Fig. S10. Wavelet coherence analysis between *R*_g_ and GPP (top) and *R*_g_ and temperature (bottom) in 2015 of 2015a (i.e. tree #1). The black contour lines indicate the 5% significance level and beyond black lines indicate the cone of influence where edge effects may distort the image. The phase difference (i.e. time lag) is shown by arrows (*R*_g_ lags behind). Arrows pointing right indicate no lag; down, ~6 h; and left, ~12 h. Refer to Table 1 for variable units.


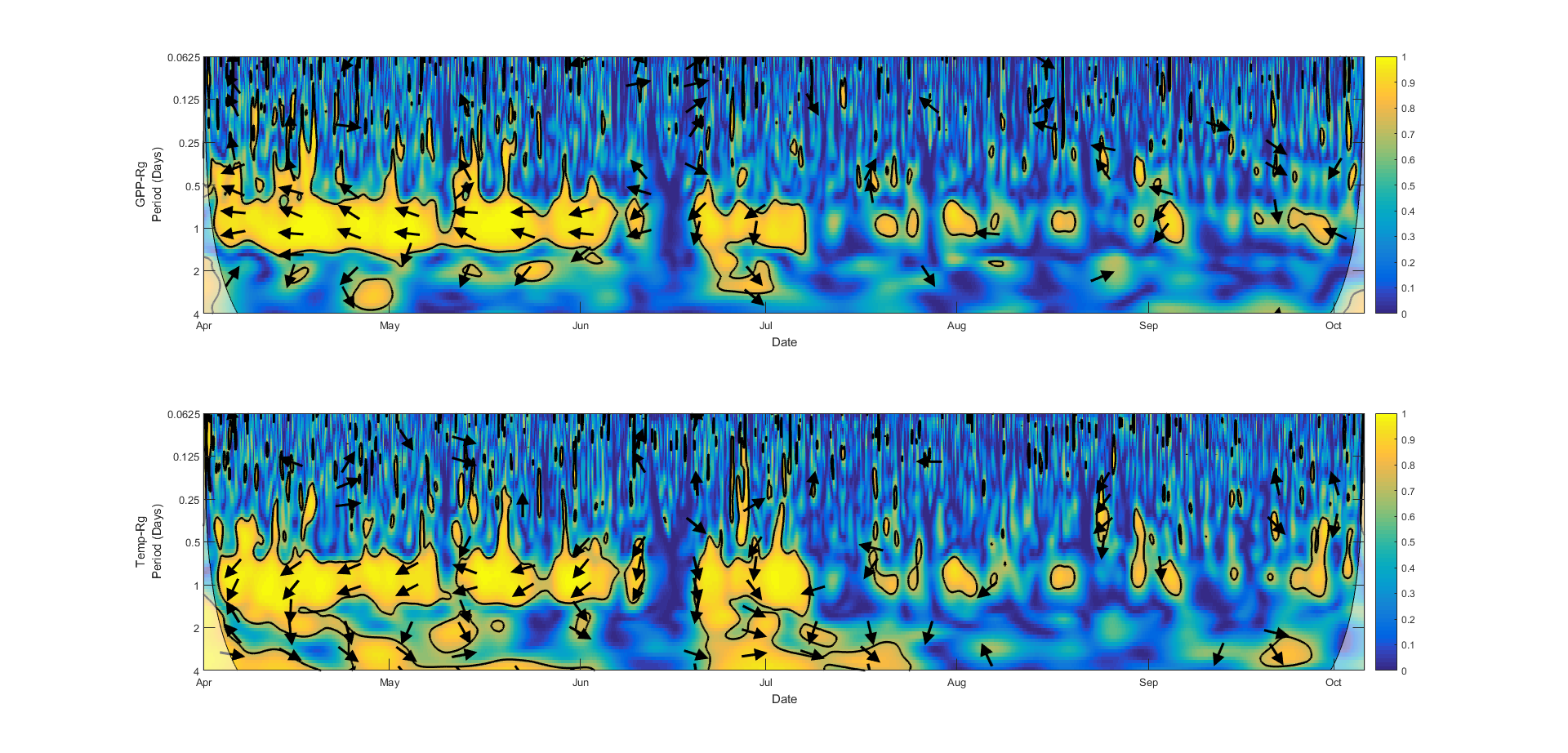


Fig. S11. Wavelet coherence analysis between *R*_g_ and GPP (top) and *R*_g_ and temperature (bottom) in 2015 of 2015b (i.e. tree #2). The black contour lines indicate the 5% significance level and beyond black lines indicate the cone of influence where edge effects may distort the image. The phase difference (i.e. time lag) is shown by arrows (*R*_g_ lags behind). Arrows pointing right indicate no lag; down, ~6 h; and left, ~12 h. Refer to Table 1 for variable units.

Table S1. Squared correlation coefficient (*r*^2^) between stem CO_2_ efflux (*E_S_*) and the estimated growth rate ($\Delta\hat{\Delta}G_{m}$). Intra-annual growth was separated into phases that represented predominant growth processes. Phase 1 is from April 1 to the date when tracheids were first observed; Phase 2 was the period from the end of Phase 1 to a day when significant decline of growth rate occurred: and Phase 3 was the period from the end of Phase 2 to October 5, when growth has stopped.

* (*P* < 0.05), ** (*P* < 0.01)

|  | Measurement | Phase 1 | Phase 2 | Phase 3 |
| --- | --- | --- | --- | --- |
| 2007 | $\Delta\hat{\Delta}G_{m}$ | 0.05* | 0.27** | 0.16** |
| 2008 | $\Delta\hat{\Delta}G_{m}$ | 0.02 | 0.36** | 0.16** |
| 2009 | $\Delta\hat{\Delta}G_{m}$ | 0.05* | 0.11** | 0.09** |
| 2011 | $\Delta\hat{\Delta}G_{m}$ | 0.00 | 0.31** | 0.00 |
| 2015a | $\Delta\hat{\Delta}G_{m}$ | 0.04 | 0.26** | 0.20** |
| 2015b | $\Delta\hat{\Delta}G_{m}$ | 0.02 | 0.23** | 0.20** |
